# Supplementary material for: Complexes of Gold(III) with Hydrazones Derived from Pyridoxal: Stability, Structure, and Nature of UV-Vis Spectra
Source: Int J Mol Sci. 2024 May 6;25(9):5046. doi: 10.3390/ijms25095046 (PMC11084471; doi:10.3390/ijms25095046)
Supplement: Supplementary file 1 [file ijms-25-05046-s001.zip › ijms-2984209-supplementary.pdf]

Supporting Information for

Complexes of Gold(III) with Hydrazones Derived from Pyridoxal: Stability, Structure, and Nature of UV-Vis Spectra

Natalia N. Kuranova, Oleg A. Pimenov, Maksim N. Zavalishin and George A. Gamov *

Department of General Chemical Technology, Ivanovo State University of Chemistry and Technology, Sheremetevskii pr. 7, Ivanovo 153000, Russia; kuranova_nn@isuct.ru (N.N.K.); pimenov@isuct.ru (O.A.P.); zavalishin00@gmail.com (M.N.Z.)
* Correspondence: ggamov@isuct.ru; Tel.: +7(915)821 85 62.

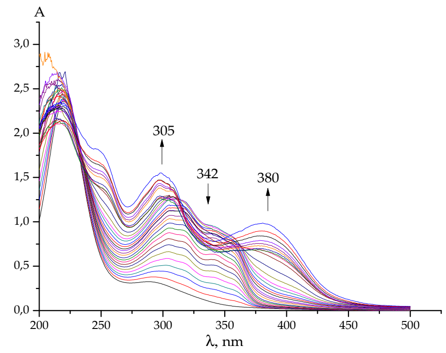

a

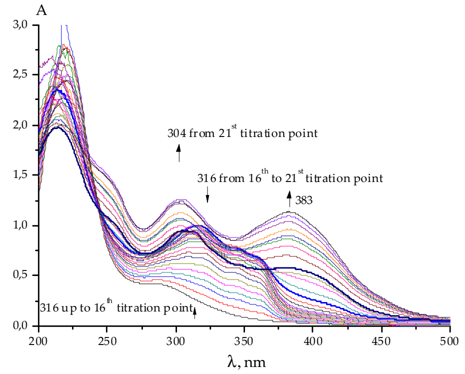

b

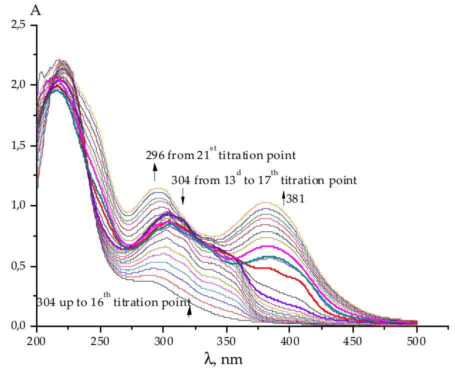

c

Figure S1. Examples of UV-Vis spectra acquired during the titration of: a) 202.4 μM $H[AuCl_4]$ + 299.4 μM $HClO_4$ by 1.433 mM **PL-T3H** + 9.928 mM NaOH; b) 202.4 μ $H[AuCl_4]$ + 299.4 μM $HClO_4$ by 1.465 mM **PL-F2H** + 10.84 mM NaOH; c) 202.4 μ $H[AuCl_4]$ + 299.4 μM $HClO_4$ by 1.642 mM **PL-F3H** + 10.80 mM NaOH in water. Initial volume 2.7 mL, 25 to 30 titration points, titrant volume = 10 μL

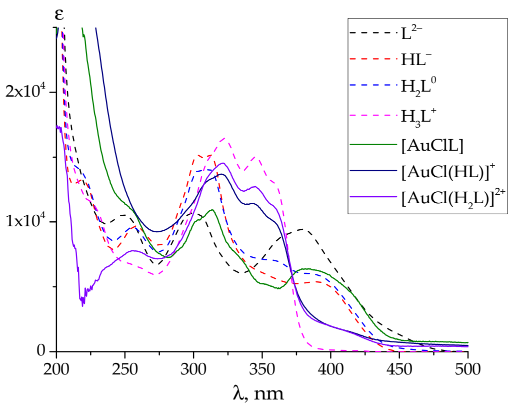

a

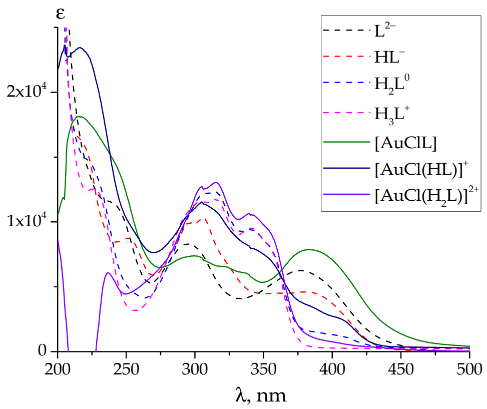

b

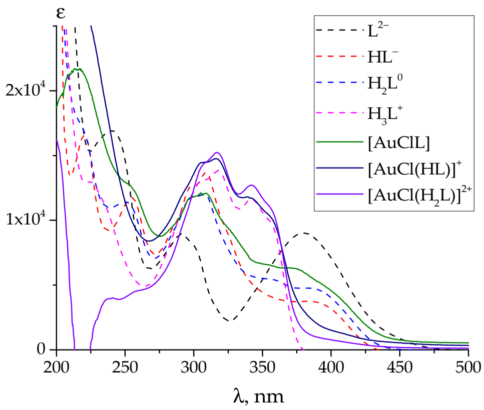

c

Figure S2. Calculated UV-Vis spectra of the individual protonated and complex species of **PL-F2H** (a), **PL-F3H** (b), **PL-T3H** (c). Spectra of the protonated species are adopted from the paper 10.1016/j.molliq.2023.123049

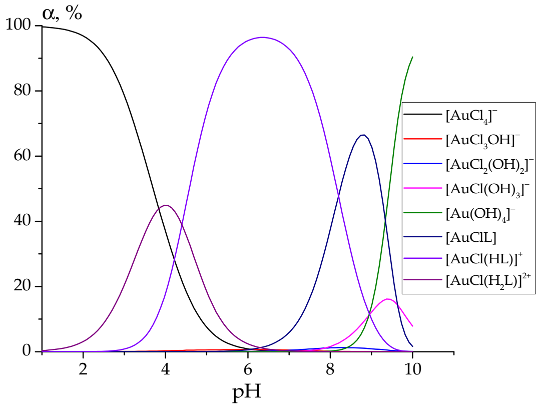

a

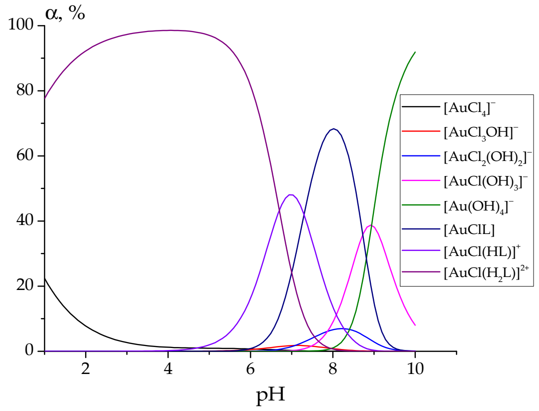

b

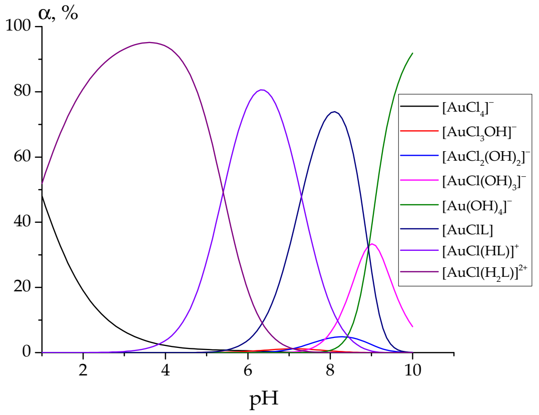

c

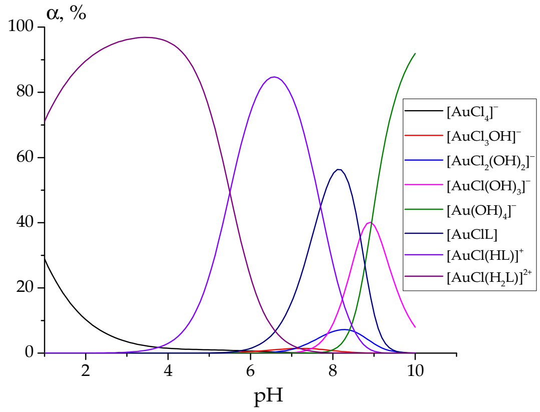

d

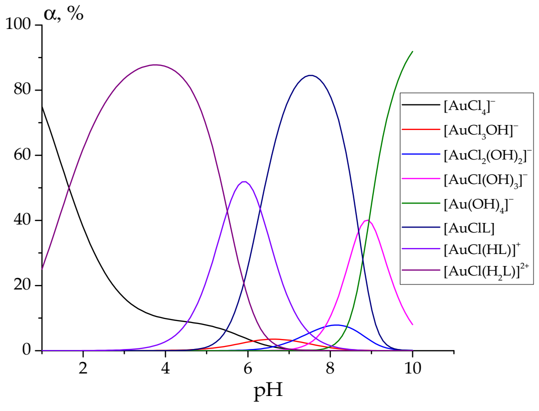

e

Figure S3. Speciation diagrams for the solutions containing 0.1 mmol L^{-1} of $H[AuCl_4]$ and 0.1 mmol L^{-1} of hydrazones: **PL-INH** (a); **PL-F2H** (b); **PL-F3H** (c); **PL-T2H** (d); **PL-T3H** (e). Charges of ionic species are omitted for clarity

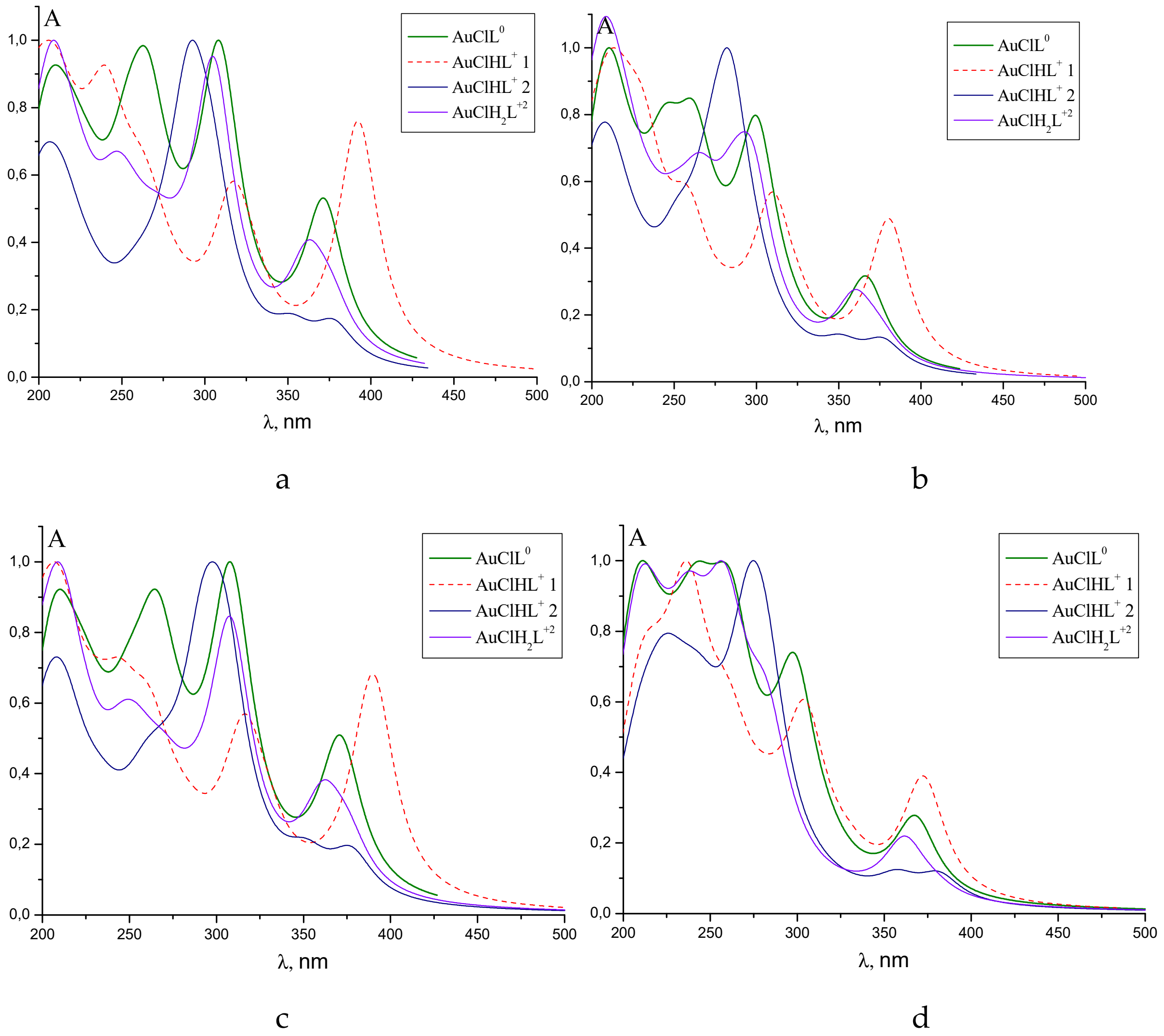

Figure S4. TD DFT simulated UV-Vis spectra of different protonated gold(III) complexes with: a) **PL-F2H**; b) **PL-T3H**; c) **PL-T2H;** d) **PL-INH** hydrazone. $AuClHL^{+}$ 1 and $AuClHL^{+}$ 2 refer to the single-protonated complexes, where proton is bound with heterocyclic nitrogen of **PL** and hydrazide nitrogen, respectively

Table S1. Optimized geometry (*xyz*-coordinates) of different protonated species of complex formed by gold(III) and hydrazone derived from pyridoxal and 2-furoylhydrazide (**PL-F2H**)

Deprotonated complex, [AuClL]

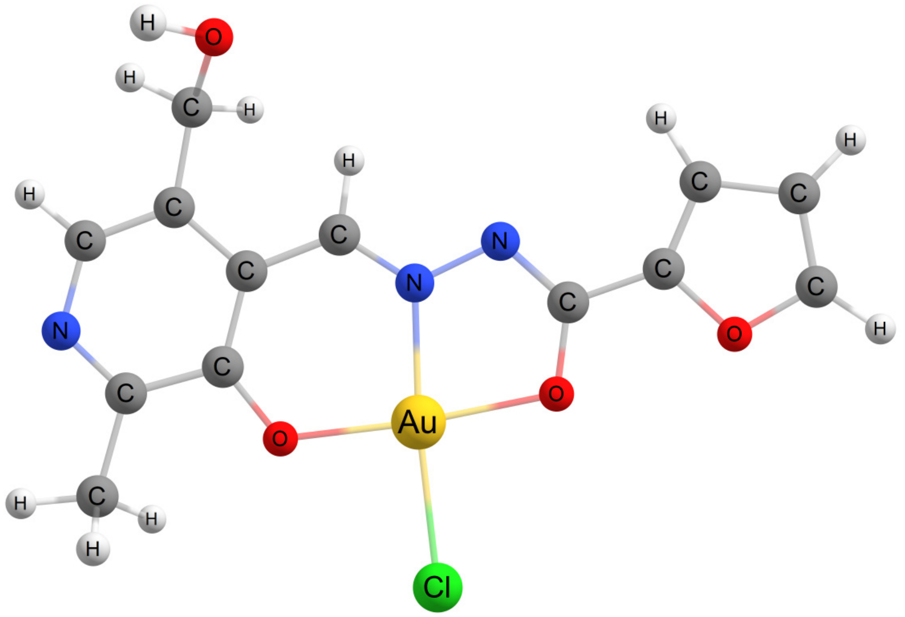

6	-3.940416000	-0.595691000	-0.040147000
6	-2.554862000	-0.232500000	-0.048270000
6	-2.219681000	1.140408000	-0.118699000
6	-3.285288000	2.083497000	-0.196916000
6	-4.575453000	1.611915000	-0.182469000
7	-4.896083000	0.307514000	-0.104891000
1	-5.405820000	2.305027000	-0.237370000
6	-4.320623000	-2.042943000	0.045812000
1	-3.918105000	-2.501645000	0.950612000
1	-3.915309000	-2.605887000	-0.796813000
1	-5.403729000	-2.131468000	0.049133000
8	-1.726701000	-1.261313000	0.013276000
6	-0.875662000	1.649115000	-0.117798000
1	-0.725184000	2.719729000	-0.129822000
6	-3.052999000	3.571399000	-0.307330000
1	-4.017109000	4.066796000	-0.437043000
1	-2.448628000	3.805444000	-1.184364000
8	-2.342647000	4.128651000	0.802577000
7	0.203786000	0.940724000	-0.076139000
7	1.440346000	1.525991000	-0.081085000
6	2.396635000	0.636944000	-0.038339000
8	2.208376000	-0.667184000	0.006490000

6	3.768447000	1.100592000	-0.040859000
6	4.322260000	2.349542000	-0.082048000
6	5.729736000	2.163510000	-0.059795000
1	3.782749000	3.278880000	-0.123036000
1	6.489383000	2.924948000	-0.080368000
8	4.753779000	0.155706000	0.005449000
6	5.934039000	0.819552000	-0.006882000
79	0.239722000	-1.046569000	0.010039000
17	0.489833000	-3.362358000	0.114320000
1	6.818243000	0.208919000	0.025663000
1	-2.857323000	3.969758000	1.601820000

Monoprotonated complex, $[AuCl(HL)]^{+}$ 1, proton is bound with heterocyclic nitrogen of pyridoxal

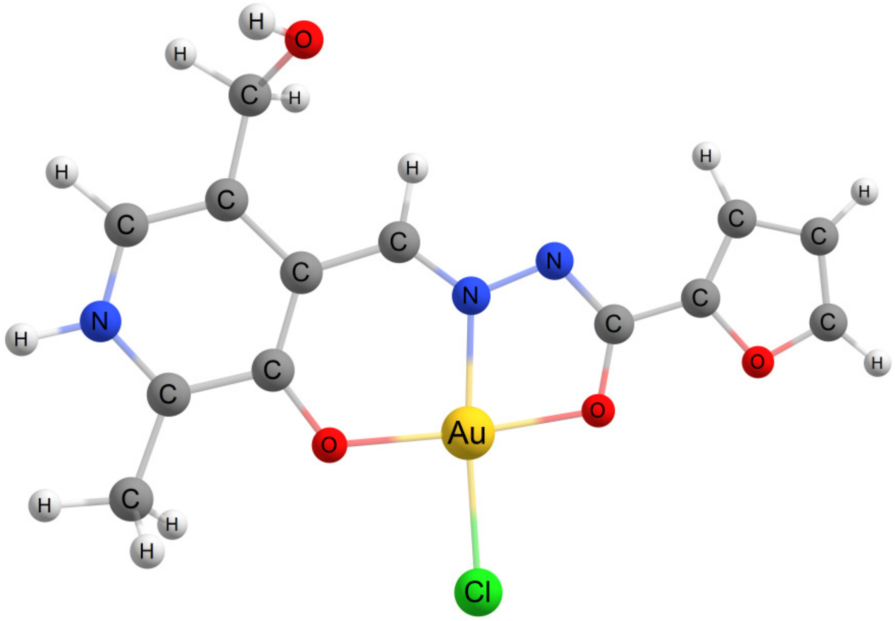

6	-3.892090000	-0.628133000	-0.047443000
6	-2.524563000	-0.243306000	-0.043976000
6	-2.200630000	1.137788000	-0.108360000
6	-3.257852000	2.088653000	-0.191864000
6	-4.552333000	1.650538000	-0.191120000
7	-4.816191000	0.327061000	-0.119032000
1	-5.404898000	2.306199000	-0.246072000
6	-4.306513000	-2.052966000	0.025549000
1	-3.918616000	-2.508718000	0.936820000
1	-3.883728000	-2.607575000	-0.812446000
1	-5.389000000	-2.150822000	0.009224000
8	-1.703565000	-1.266016000	0.020645000
6	-0.853727000	1.653908000	-0.103685000
1	-0.706411000	2.724031000	-0.109689000
6	-3.016129000	3.581109000	-0.295560000
1	-3.976431000	4.085189000	-0.410278000

1	-2.427074000	3.801410000	-1.185611000
8	-2.283803000	4.111929000	0.802517000
7	0.224264000	0.942766000	-0.066958000
7	1.449654000	1.523347000	-0.073159000
6	2.412815000	0.631563000	-0.035751000
8	2.221658000	-0.669934000	0.008253000
6	3.777228000	1.096493000	-0.044988000
6	4.324689000	2.350486000	-0.090908000
6	5.730395000	2.171751000	-0.076579000
1	3.779486000	3.276586000	-0.130128000
1	6.486435000	2.936403000	-0.102521000
8	4.767644000	0.156659000	-0.003336000
6	5.941480000	0.827162000	-0.023269000
79	0.264466000	-1.049731000	0.014909000
17	0.518116000	-3.354868000	0.114325000
1	6.830042000	0.222506000	0.004730000
1	-2.818371000	4.033172000	1.600834000
1	-5.788397000	0.039519000	-0.119022000

Monoprotonated complex, $[AuCl(HL)]^{+}$ 2, proton is bound with hydrazide nitrogen

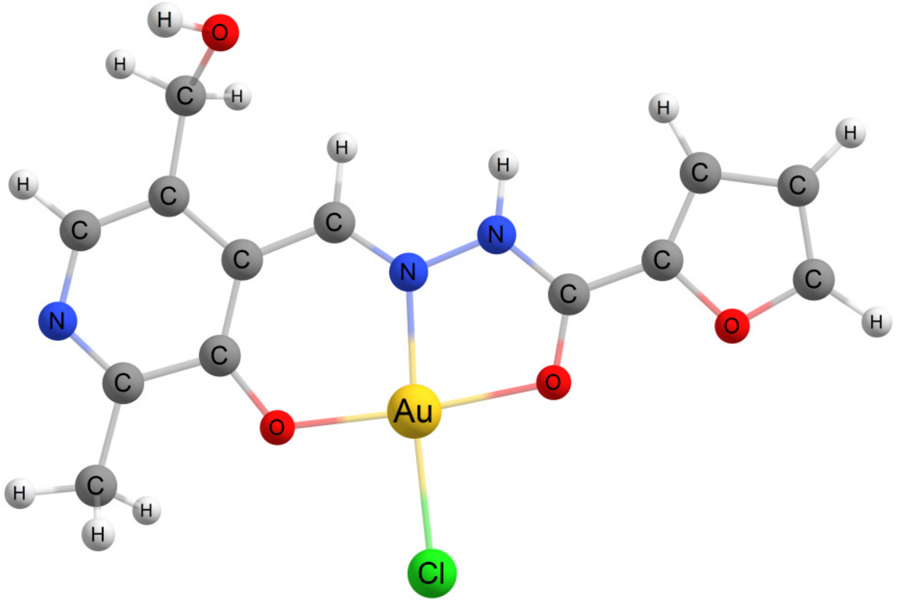

6	-3.945657000	-0.604721000	-0.028583000
6	-2.560045000	-0.228363000	-0.039595000
6	-2.239777000	1.149238000	-0.106328000
6	-3.311412000	2.087670000	-0.181633000
6	-4.595120000	1.604172000	-0.163184000
7	-4.900280000	0.295486000	-0.087179000
1	-5.433408000	2.287254000	-0.213170000
6	-4.315434000	-2.053511000	0.055052000
1	-3.908710000	-2.509704000	0.959048000
1	-3.907074000	-2.610726000	-0.789652000

1	-5.397708000	-2.148149000	0.059412000
8	-1.735704000	-1.260667000	0.018775000
6	-0.902499000	1.668997000	-0.100907000
1	-0.776875000	2.744926000	-0.093730000
6	-3.090520000	3.577298000	-0.310636000
1	-4.062356000	4.070228000	-0.362843000
1	-2.561747000	3.805518000	-1.236848000
8	-2.289830000	4.138286000	0.731980000
7	0.159215000	0.939175000	-0.067621000
7	1.431135000	1.460928000	-0.063624000
6	2.454285000	0.591611000	-0.033001000
8	2.216356000	-0.666710000	-0.000369000
6	3.801304000	1.066014000	-0.035569000
6	4.371898000	2.315722000	-0.068150000
6	5.767968000	2.111947000	-0.049070000
1	3.863764000	3.264256000	-0.101075000
1	6.535828000	2.864394000	-0.064684000
8	4.776719000	0.109726000	0.002131000
6	5.954409000	0.760176000	-0.006547000
79	0.221163000	-1.054212000	0.007521000
17	0.449287000	-3.342476000	0.098311000
1	6.834337000	0.142624000	0.020288000
1	-2.747993000	4.016312000	1.571302000
1	1.545001000	2.466173000	-0.095735000

Bis-protonated complex, $[AuCl(H_2L)]^{2+}$

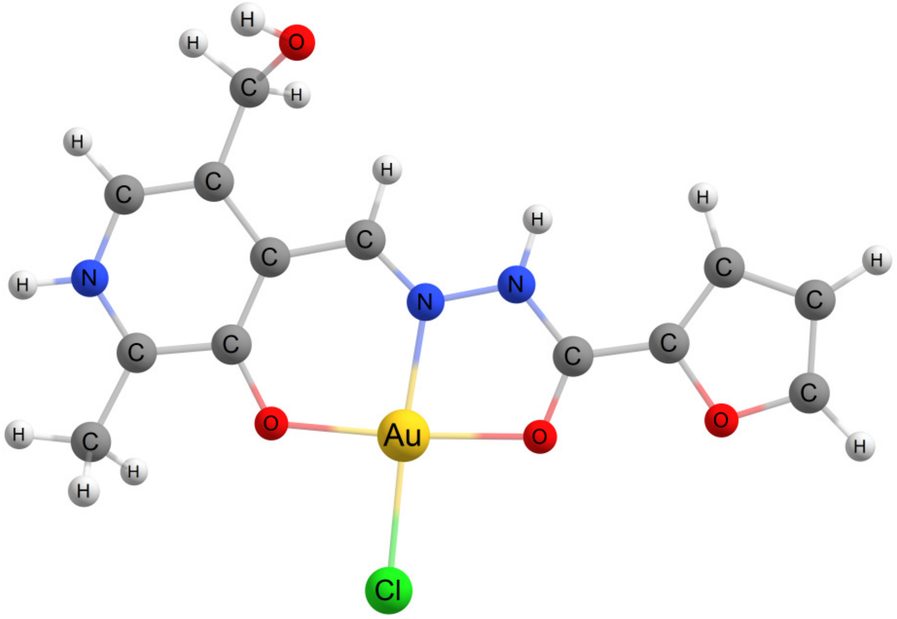

6	-3.897832000	-0.646713000	-0.035397000
6	-2.530256000	-0.243381000	-0.036304000
6	-2.228321000	1.140764000	-0.096741000
6	-3.293346000	2.083179000	-0.175998000

6	-4.581270000	1.627841000	-0.170037000
7	-4.826106000	0.299926000	-0.099676000
1	-5.443296000	2.271371000	-0.218270000
6	-4.294122000	-2.075335000	0.035404000
1	-3.894420000	-2.528132000	0.943072000
1	-3.868762000	-2.620835000	-0.807340000
1	-5.375164000	-2.185499000	0.025018000
8	-1.708846000	-1.266919000	0.022527000
6	-0.883982000	1.674396000	-0.089328000
1	-0.767947000	2.750827000	-0.077098000
6	-3.071702000	3.579468000	-0.295571000
1	-4.041923000	4.075395000	-0.319169000
1	-2.572381000	3.800098000	-1.239102000
8	-2.241264000	4.115622000	0.725410000
7	0.175121000	0.947659000	-0.060492000
7	1.440874000	1.467433000	-0.058142000
6	2.468002000	0.594452000	-0.031337000
8	2.225879000	-0.664627000	0.002015000
6	3.810282000	1.063942000	-0.039306000
6	4.383686000	2.315303000	-0.072932000
6	5.776648000	2.109550000	-0.062145000
1	3.876880000	3.264772000	-0.101079000
1	6.546025000	2.860275000	-0.080842000
8	4.785435000	0.106274000	-0.008746000
6	5.961227000	0.755496000	-0.023092000
79	0.248315000	-1.050517000	0.011717000
17	0.487839000	-3.328083000	0.101256000
1	6.841523000	0.137987000	-0.002639000
1	-2.703879000	4.064756000	1.569827000
1	1.556568000	2.473230000	-0.089710000
1	-5.796295000	0.002338000	-0.094415000

Table S2. Calculated IR spectra of different protonated species of complex formed by gold(III) and hydrazone derived from pyridoxal and 2-furoylhydrazide (**PL-F2H**)

Deprotonated complex, [AuClL]		Monoprotonated complex 1, $[AuCl(HL)]^+$		Monoprotonated complex 2, $[AuCl(HL)]^+$		*Bis*-protonated complex, $[AuCl(H_2L)]^{2+}$	
Frequency, cm^{-1}	Relative intensity	Frequency, cm^{-1}	Relative intensity	Frequency, cm^{-1}	Relative intensity	Frequency, cm^{-1}	Relative intensity
30.9063	0.559	31.6196	9.0936	29.5429	0.5152	29.5591	9.8421
40.3515	0.5533	43.3067	0.043	40.0332	5	46.4094	3.3321
48.6975	0.0628	54.4983	0.0264	66.2121	0.5052	66.7537	5.1087
67.171	2.4306	68.1503	4.662	67.8859	4.3262	72.5748	0.4046
78.3733	1.4466	81.5881	4.062	77.4903	0.7424	78.0745	1.7599
96.5272	1.1377	98.9113	0.037	94.5551	2.2331	98.9013	0.0462
104.4718	4.5564	104.3481	2.1072	108.0259	0.306	111.4337	0.3255
109.8207	1.8888	110.896	4.2337	114.3358	4.0365	116.0604	4.696
118.1998	0.757	121.339	0.3243	122.3615	6.4928	125.5158	2.1967
154.324	0.1338	139.173	0.7351	156.3968	0.6611	145.2937	1.0257
175.5502	11.0898	177.0801	4.656	172.8465	5.0638	175.5896	2.8129
186.5855	2.2779	184.8734	4.1538	187.9078	5.0746	189.9034	7.2125
192.9122	1.0332	192.1447	1.689	191.2357	3.2502	193.5812	3.8668
203.5387	0.316	208.4859	2.2278	201.0388	1.1226	205.6251	4.6264
225.2466	0.8611	223.0042	2.1451	222.4465	1.5244	221.4719	1.9307
242.8203	6.9884	244.9556	4.9214	238.1258	13.3445	242.2587	8.0653
255.2082	1.5517	255.0745	0.7284	246.8971	2.8593	250.7354	2.5879
276.238	0.7805	272.3225	3.1098	271.8125	0.4423	272.2925	4.835
310.5977	145.4232	303.5913	143.3149	318.8155	88.0486	320.1907	69.1188
325.9656	70.3303	333.7867	72.8964	339.7339	12.4608	343.9318	17.2042
343.3474	9.414	341.1857	11.3567	347.6232	68.0174	355.5864	64.9961
363.3928	72.9489	361.3273	54.9447	366.4191	110.2674	369.0654	107.8513
402.0155	4.0727	396.4765	8.852	384.8483	9.3023	391.8255	9.2986
420.6409	18.4159	422.0359	14.1356	397.8193	4.5132	411.6685	15.3945
437.4553	1.4548	444.4831	27.6537	417.0992	9.9498	420.5255	5.6547
448.8285	32.8095	459.8243	1.9041	447.7846	37.6632	444.4936	31.1922
500.5801	48.0072	500.0449	26.9701	495.9189	61.4053	496.0544	42.4439
531.8029	9.1613	529.6046	16.4355	506.6061	122.9213	529.8211	19.9491
572.4848	7.0075	567.3495	1.7604	530.5088	13.3756	533.7375	117.4133
578.089	26.1149	571.1001	8.0647	565.3865	3.8328	565.4614	6.7506
608.9033	13.0561	605.9048	13.8298	572.7417	30.7303	565.8408	3.3341
613.5277	22.3315	610.3448	5.7906	598.8214	22.0132	595.8743	24.4302
631.3507	7.5212	630.5642	9.283	607.721	40.0111	605.182	10.7711
647.0861	70.0654	654.5867	31.5069	621.0432	2.6598	619.5525	3.7922
659.5009	6.6962	662.6793	16.7933	633.5853	95.2682	645.27	64.4373
685.1941	39.7707	686.7514	31.6035	651.9094	9.4865	655.7829	17.5174
742.6717	16.7236	744.7955	21.1513	683.4624	15.004	685.3866	11.6765
758.6671	1.1301	760.4113	4.5855	738.1838	12.5415	738.5011	13.4996
776.2125	5.046	775.3475	0.6978	757.9423	2.4108	760.1636	7.9897
776.8152	87.6965	783.7238	83.2779	774.2982	5.5928	777.8198	7.331

804.041	2.6954	791.5785	47.7292	797.064	80.7766	796.1056	45.5832
865.4117	5.4559	834.8928	77.8165	802.487	1.6893	801.2793	75.8319
896.8933	8.3079	873.1661	3.4078	887.7967	10.5331	841.5034	89.2033
903.6966	81.6581	896.6011	10.3728	891.2787	0.7219	887.9111	7.3314
918.5819	30.0282	903.5305	128.3822	903.1767	76.0266	899.2666	0.303
922.2454	1.0586	917.2712	2.2662	920.1467	29.5146	902.9448	93.3631
949.7814	7.5754	929.0635	1.0079	942.9331	1.033	926.2232	2.3839
957.4826	36.5924	936.8241	11.4182	958.4159	87.9381	949.0749	0.9768
966.2128	23.9467	960.4489	29.9292	964.8179	60.3158	959.6624	114.6978
988.727	69.7237	967.9068	28.6803	975.7674	11.807	963.73	39.596
1018.489	69.1009	1001.078	87.3727	988.4258	68.0195	976.8279	28.6437
1026.313	224.2808	1028.087	279.2122	1027.283	85.1703	1004.165	41.6419
1040.158	14.8511	1040.019	125.3869	1043.358	47.1924	1042.398	37.8632
1054.145	1.7612	1047.562	51.4205	1054.753	1.7443	1050.834	147.009
1072.418	13.9833	1057.277	8.2553	1063.598	106.9392	1058.151	8.9463
1092.596	18.0448	1083.166	22.7247	1071.507	20.9596	1066.876	97.3231
1107.767	37.0227	1094.905	80.8149	1120.032	7.4618	1080.033	39.5717
1156.261	231.759	1112.009	28.5668	1123.368	251.3335	1119.979	66.2616
1198.588	16.5949	1157.753	517.9159	1174.412	197.8954	1126.653	282.9321
1202.907	41.8624	1207.086	9.458	1198.828	221.6494	1175.885	280.2056
1216.871	389.5201	1208.682	110.7025	1206.025	63.5652	1208.929	16.5549
1258.375	25.8794	1225.193	309.2734	1224.773	135.2969	1214.561	182.3495
1290.015	199.5721	1258.566	26.616	1278.832	5.7418	1226.338	225.5993
1316.161	22.3758	1269.052	262.9479	1293.444	219.0745	1264.398	166.8084
1324.065	94.2244	1325.043	47.9886	1311.59	66.4654	1280.27	20.9721
1349.312	289.3315	1331.262	7.9785	1322.491	178.5518	1317.018	71.9504
1371.374	11.7456	1363.318	384.6089	1367.629	170.9493	1337.887	124.5318
1391.266	142.3049	1376.162	11.09	1376.045	53.7396	1375.059	111.3084
1405.466	102.6694	1392.241	232.7108	1387.523	19.9571	1385.522	77.8528
1410.798	34.472	1408.054	70.8455	1407.789	43.0056	1390.328	122.4926
1414.736	12.5952	1412.906	72.8939	1411.009	28.7944	1407.127	45.0394
1425.913	42.9504	1422.131	2.1644	1415.182	150.0294	1416.478	223.8847
1464.805	26.0514	1430.176	11.5851	1436.763	255.6931	1420.696	51.4897
1466.325	11.0531	1439.332	99.4338	1453.502	288.8823	1434.91	38.4242
1484.404	10.9298	1451.583	17.8742	1464.567	2.8703	1439.584	360.7056
1500.631	407.2516	1481.175	693.7811	1466.763	11.826	1450.298	21.6806
1513.264	9.1683	1489.569	1254.312	1483.861	35.829	1452.92	363.5834
1541.662	725.0253	1505.228	111.8033	1505.36	343.2988	1483.816	12.6279
1554.11	210.0614	1512.739	77.6044	1510.821	101.425	1501.317	226.7291
1598.222	8.2076	1531.51	639.8777	1545.637	247.041	1509.531	143.6442
1610.634	147.4035	1594.953	234.629	1579.134	190.2428	1522.978	341.0862
1653.089	206.9368	1620.71	56.5201	1600.435	47.5807	1573.417	292.6501
3035.148	39.9836	1648.832	54.7368	1626.91	1254.479	1617.482	56.7048
3035.423	57.2428	1668.63	72.4297	1669.433	47.3854	1628.222	1451.272
3076.854	28.1347	3047.789	56.4864	3037.93	15.2904	1660.862	51.8355
3080.862	17.4811	3049.229	2.4257	3041.923	60.6501	1683.86	10.1027

3133.908	23.3477	3091.514	17.3074	3080.499	25.7251	3050.744	2.1252
3161.755	47.6283	3103.281	0.8211	3084.879	13.7104	3053.772	39.9093
3198.379	31.5773	3138.598	17.9205	3138.778	18.7067	3094.392	17.6479
3253.734	3.9355	3208.986	49.277	3167.818	73.857	3104.816	0.1307
3267.55	1.5223	3248.764	2.198	3169.603	34.9058	3144.149	13.6894
3281.182	0.3164	3256.003	3.111	3256.562	0.5933	3172.436	111.1685
3792.404	67.3498	3268.962	3.1064	3267.231	5.4447	3249.806	6.1371
		3281.96	0.5824	3284.748	3.38	3257.716	0.6859
		3537.081	406.4443	3560.419	161.4264	3268.453	6.7293
		3793.259	89.1798	3791.708	76.7772	3285.064	4.4924
						3528.84	351.907
						3551.656	177.1162
						3789.851	98.8847

Table S3. Calculated TD-DFT spectra of different protonated species of complex formed by gold(III) and hydrazone derived from pyridoxal and 2-furoylhydrazide (**PL-F2H**)

Deprotonated complex, [AuClL]		Monoprotonated complex 1, $[AuCl(HL)]^{+}$		Monoprotonated complex 2, $[AuCl(HL)]^{+}$		*Bis*-protonated complex, $[AuCl(H_2L)]^{2+}$	
λ, nm	oscillator strength	λ, nm	oscillator strength	λ, nm	oscillator strength	λ, nm	oscillator strength
481.54	0	449.7	0.0001	525.41	0	454.34	0
371.98	0.1136	392.34	0.5588	377.82	0.117	376.12	0.1057
371.51	0.2062	347.93	0.0001	367.47	0.0002	366.31	0
317.45	0.0298	328.16	0.0756	352.67	0.0803	362.1	0.2309
313	0.0003	327.97	0.0075	327.04	0.0001	320.74	0.0001
308.44	0.5742	316.57	0.3157	308.7	0.049	305.19	0.7245
292.77	0.0003	294.84	0.0001	303.51	0.3588	299.29	0
291.43	0.0028	283.82	0.0348	301.98	0.0007	285.41	0.0392
279.46	0.0363	269.28	0.0684	290.76	0.7339	271.77	0.1329
265.51	0.3924	268.02	0.0674	276.71	0.1063	261.3	0.062
253.08	0.2161	259.42	0.1633	265.36	0.0214	252.33	0.1293
249.19	0.0026	249.82	0.0048	259.43	0.0805	245.34	0.1845
243.24	0.0015	241.25	0.3837	252.17	0.0286	243.06	0.0066
237.41	0.02	236.29	0.009	237.33	0.0062	236.76	0.005
230.47	0.0605	230.7	0.0002	232.98	0.0687	229.62	0.0144
228.2	0.053	230.17	0.0923	224.46	0.0321	225.99	0.1049
221.9	0.0827	228.83	0.0065	219.91	0.0802	223.28	0.0006
216.73	0.0709	221.08	0.0536	218.58	0.0033	220.92	0.0085
214.46	0.052	219.67	0.0022	217	0.0575	219.78	0.0572
213.14	0.005	216.11	0.0037	215.73	0.0026	217.95	0.0309
209.78	0.0834	215.93	0.0581	212.35	0.0131	210.47	0.0093
208.55	0.0157	211.21	0.1484	211.91	0.1506	209.79	0.3714
207.29	0.0035	208.71	0.0323	210.5	0.0056	208.4	0.0272
206.29	0.0013	206.54	0.0152	209.39	0.0927	204.51	0.201
206.04	0.088	206.25	0.1374	204.41	0.0229	196.11	0.0051
204.52	0.0009	198.45	0.0447	203.86	0.01	195.48	0.0073
203.54	0.1314	197.53	0.0253	202.09	0.1134	194.78	0.02
199.68	0.0668	195.77	0.1941	199.03	0.1638	193.2	0.1142
195.81	0.0021	191.21	0.1487	196.14	0.0522	191.2	0.0416
193.92	0.0566	190.59	0.0138	195.74	0.0827	189.8	0.0052

Table S4. Optimized geometry (*xyz*-coordinates) of different protonated species of complex formed by gold(III) and hydrazone derived from pyridoxal and 3-furoylhydrazide (**PL-F3H**)

Deprotonated complex, [AuClL]

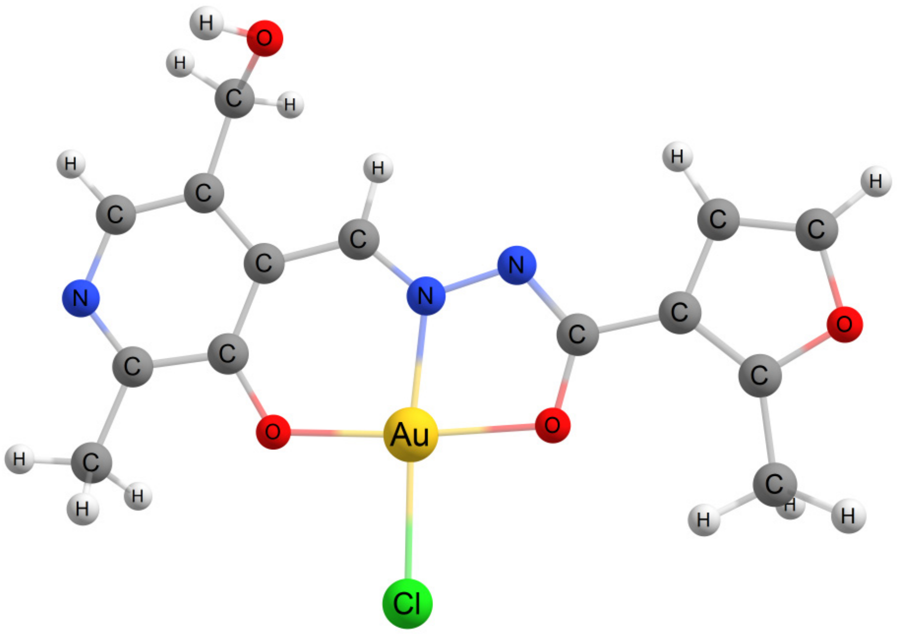

6	-4.108948000	-0.624423000	-0.041887000
6	-2.726044000	-0.250842000	-0.048903000
6	-2.401628000	1.125060000	-0.112422000
6	-3.474524000	2.060407000	-0.182272000
6	-4.761128000	1.579039000	-0.168042000
7	-5.071772000	0.271688000	-0.098960000
1	-5.596804000	2.266259000	-0.216231000
6	-4.477781000	-2.075217000	0.034218000
1	-4.071108000	-2.537158000	0.935511000
1	-4.068465000	-2.629054000	-0.812513000
1	-5.560155000	-2.172385000	0.037427000
8	-1.889495000	-1.272629000	0.006821000
6	-1.061095000	1.644156000	-0.113232000
1	-0.919424000	2.716019000	-0.121885000
6	-3.254050000	3.550827000	-0.282670000
1	-4.222261000	4.039576000	-0.407079000
1	-2.653178000	3.795995000	-1.159078000
8	-2.546112000	4.105757000	0.830001000
7	0.024411000	0.945744000	-0.077499000
7	1.256633000	1.544026000	-0.082217000
6	2.228309000	0.670175000	-0.044147000
8	2.038077000	-0.637829000	-0.002876000

6	3.596991000	1.156197000	-0.050203000
6	3.988281000	2.540887000	-0.136108000
6	5.333494000	2.553094000	-0.112374000
1	3.332088000	3.389207000	-0.206160000
1	6.072221000	3.332293000	-0.151529000
6	4.757604000	0.424172000	0.021554000
6	5.088099000	-1.016555000	0.124976000
1	4.185531000	-1.607243000	0.231077000
1	5.625001000	-1.350736000	-0.765583000
1	5.736612000	-1.195037000	0.984502000
8	5.815283000	1.272592000	-0.015951000
79	0.076799000	-1.038768000	0.003119000
17	0.350500000	-3.353952000	0.100707000
1	-3.058083000	3.936947000	1.628944000

Monoprotonated complex, $[AuCl(HL)]^{+}$ 1, proton is bound with heterocyclic nitrogen of pyridoxal

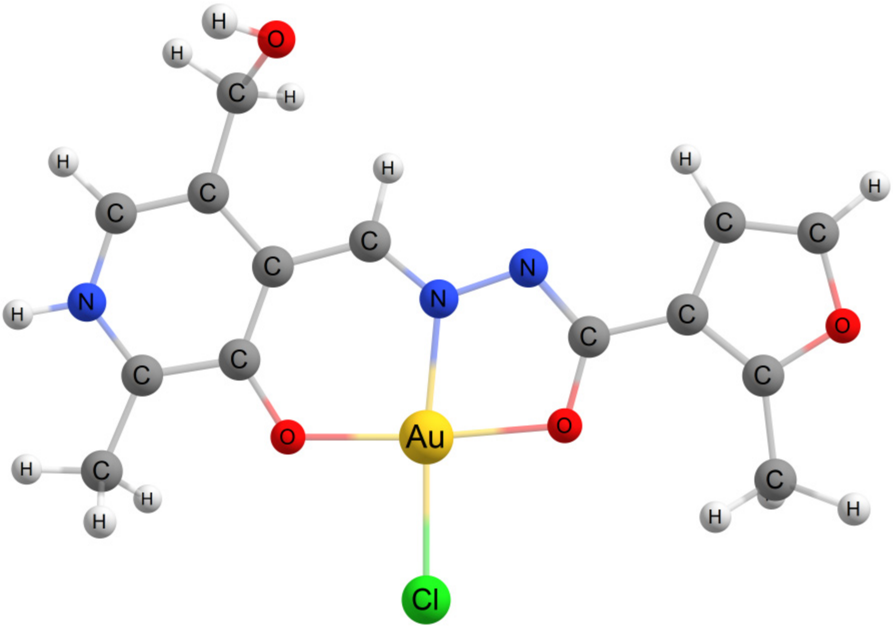

6	-4.059094000	-0.661989000	-0.047064000
6	-2.694895000	-0.264107000	-0.043509000
6	-2.384832000	1.120514000	-0.100993000
6	-3.451222000	2.061645000	-0.174922000
6	-4.741583000	1.611251000	-0.172717000
7	-4.992315000	0.284726000	-0.109095000
1	-5.600597000	2.258993000	-0.220041000
6	-4.458923000	-2.091455000	0.016089000
1	-4.063290000	-2.550372000	0.922411000
1	-4.032995000	-2.634988000	-0.827581000
1	-5.540376000	-2.200601000	0.002323000
8	-1.863806000	-1.278062000	0.014477000
6	-1.042162000	1.649547000	-0.098580000

1	-0.905683000	2.721154000	-0.100623000
6	-3.224575000	3.557133000	-0.269016000
1	-4.190364000	4.052427000	-0.375921000
1	-2.641712000	3.789709000	-1.160037000
8	-2.492780000	4.087280000	0.829731000
7	0.042936000	0.950382000	-0.068245000
7	1.263123000	1.546488000	-0.072173000
6	2.243277000	0.671759000	-0.042234000
8	2.053026000	-0.634154000	-0.006554000
6	3.604453000	1.159610000	-0.053747000
6	3.996848000	2.542843000	-0.158129000
6	5.341383000	2.552040000	-0.137119000
1	3.342074000	3.391365000	-0.237464000
1	6.082743000	3.327959000	-0.186202000
6	4.766065000	0.424976000	0.026089000
6	5.089734000	-1.015951000	0.139306000
1	4.215052000	-1.583859000	0.436166000
1	5.444891000	-1.405937000	-0.817830000
1	5.884722000	-1.163423000	0.870560000
8	5.820665000	1.269915000	-0.023803000
79	0.103881000	-1.039145000	0.005878000
17	0.384904000	-3.343075000	0.096652000
1	-3.022604000	3.996407000	1.629910000
1	-5.961699000	-0.012007000	-0.108054000

Monoprotonated complex, $[AuCl(HL)]^+$ 2, proton is bound with hydrazide nitrogen

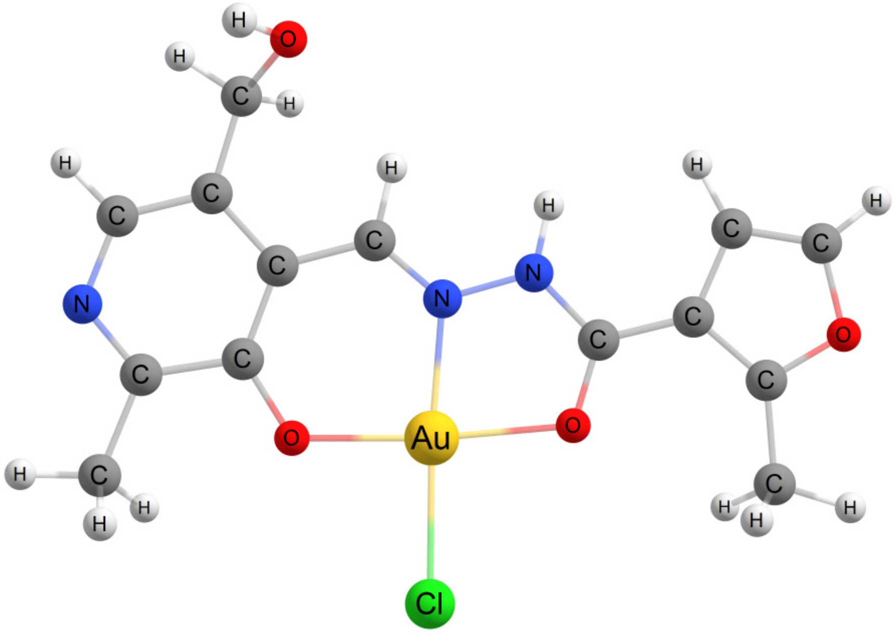

6	-4.104376000	-0.660099000	-0.038371000
6	-2.725449000	-0.259899000	-0.043942000
6	-2.429499000	1.123514000	-0.100866000

6	-3.517166000	2.043882000	-0.166239000
6	-4.792372000	1.538272000	-0.150989000
7	-5.074815000	0.223731000	-0.088319000
1	-5.642312000	2.207355000	-0.192976000
6	-4.448979000	-2.115951000	0.029779000
1	-4.036520000	-2.574274000	0.930087000
1	-4.028915000	-2.657348000	-0.819471000
1	-5.529441000	-2.229589000	0.030333000
8	-1.882625000	-1.277182000	0.009539000
6	-1.101236000	1.666171000	-0.097145000
1	-0.993865000	2.743877000	-0.085752000
6	-3.321287000	3.538197000	-0.278409000
1	-4.301160000	4.015289000	-0.328436000
1	-2.793059000	3.786081000	-1.199902000
8	-2.533724000	4.100140000	0.773759000
7	-0.026833000	0.955485000	-0.071820000
7	1.236616000	1.498345000	-0.068477000
6	2.281239000	0.654161000	-0.042053000
8	2.057360000	-0.610349000	-0.008716000
6	3.615796000	1.162962000	-0.050919000
6	4.056213000	2.534674000	-0.133372000
6	5.398653000	2.489567000	-0.106134000
1	3.459825000	3.427280000	-0.205855000
1	6.169124000	3.236650000	-0.142625000
6	4.755370000	0.381861000	0.022227000
6	4.999485000	-1.073880000	0.123313000
1	4.461576000	-1.498331000	0.970355000
1	4.648373000	-1.587474000	-0.772584000
1	6.063928000	-1.258606000	0.243338000
8	5.827831000	1.185848000	-0.010671000
79	0.071811000	-1.034461000	0.001608000
17	0.342004000	-3.318955000	0.095028000
1	-2.991365000	3.957416000	1.610102000
1	1.332786000	2.505173000	-0.091338000

Bis-protonated complex, $[AuCl(H_2L)]^{2+}$

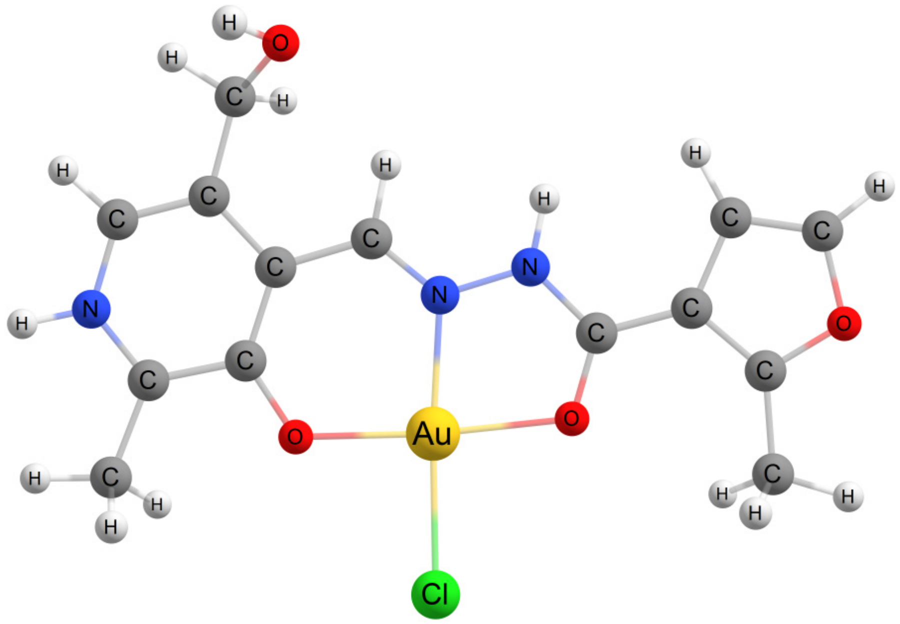

6	-4.054907000	-0.702993000	-0.043078000
6	-2.694679000	-0.275201000	-0.041482000
6	-2.418401000	1.114616000	-0.093967000
6	-3.500299000	2.038340000	-0.162445000
6	-4.779947000	1.560228000	-0.156078000
7	-5.000148000	0.227323000	-0.097005000
1	-5.653685000	2.188282000	-0.196059000
6	-4.424924000	-2.139340000	0.014157000
1	-4.014193000	-2.594049000	0.915886000
1	-3.991888000	-2.668088000	-0.835350000
1	-5.503786000	-2.269830000	0.005492000
8	-1.854512000	-1.283117000	0.011038000
6	-1.083680000	1.672237000	-0.089482000
1	-0.986594000	2.750318000	-0.074790000
6	-3.304200000	3.539074000	-0.267911000
1	-4.282687000	4.018678000	-0.291682000
1	-2.803776000	3.777231000	-1.206616000
8	-2.487830000	4.078923000	0.762562000
7	-0.011496000	0.965342000	-0.066692000
7	1.245645000	1.506886000	-0.065697000
6	2.294939000	0.659970000	-0.039591000
8	2.067685000	-0.605346000	-0.004111000
6	3.624919000	1.164613000	-0.051384000
6	4.070956000	2.535639000	-0.126189000
6	5.412353000	2.483722000	-0.106536000
1	3.478900000	3.431786000	-0.187966000
1	6.186922000	3.226521000	-0.141472000
6	4.764511000	0.376593000	0.009812000
6	5.000162000	-1.080126000	0.099124000
1	4.482189000	-1.502501000	0.959943000

1	4.618029000	-1.586944000	-0.787857000
1	6.065829000	-1.273712000	0.188591000
8	5.836344000	1.175399000	-0.023114000
79	0.099937000	-1.029151000	0.005882000
17	0.383037000	-3.302259000	0.101794000
1	-2.952890000	4.009598000	1.604339000
1	-5.964453000	-0.088228000	-0.092368000
1	1.343099000	2.514326000	-0.090249000

Table S5. Calculated IR spectra of different protonated species of complex formed by gold(III) and hydrazone derived from pyridoxal and 3-furoylhydrazide (**PL-F3H**)

Deprotonated complex, [AuClL]		Monoprotonated complex 1, $[AuCl(HL)]^+$		Monoprotonated complex 2, $[AuCl(HL)]^+$		*Bis*-protonated complex, $[AuCl(H_2L)]^{2+}$	
Frequency, cm^{-1}	Relative intensity	Frequency, cm^{-1}	Relative intensity	Frequency, cm^{-1}	Relative intensity	Frequency, cm^{-1}	Relative intensity
21.7995	0.1798	18.9578	0.7969	27.9933	0.0441	27.7333	5.3077
30.2817	0.1505	30.4837	7.1654	32.9847	0.2329	32.7365	2.2158
41.3928	0.3682	34.4767	0.6491	46.4148	4.6036	47.2952	2.5927
54.5279	0.9282	48.7126	0.1779	61.6914	0.2516	61.7632	1.4984
66.3111	1.253	70.1267	3.3311	72.9984	2.764	74.9989	5.848
77.4295	2.1421	81.7726	4.9594	78.3385	1.7611	83.4778	0.1734
94.0923	0.4136	96.8356	0.3616	95.595	0.8129	99.8232	0.6348
105.1605	3.1384	104.6679	1.1896	109.2858	1.0848	110.2526	1.1961
109.1253	1.7502	109.7963	4.0891	113.0815	3.5542	113.8002	4.4103
119.1922	1.1826	122.4276	0.3299	122.7276	9.4258	124.9136	4.3277
154.8682	0.1269	140.1484	0.8423	156.1998	0.6225	146.849	1.3825
171.4796	2.6564	172.7636	1.6698	162.8152	4.3196	163.8765	3.1525
177.7066	5.9878	179.4812	2.9151	175.534	3.0273	177.3716	1.0472
190.0989	0.1628	188.2438	1.2891	187.5299	1.0085	189.3884	3.094
201.2031	0.3552	204.9113	1.1586	198.6821	1.6765	201.4098	3.39
212.4903	1.0414	211.9996	1.434	210.9777	3.0595	212.1738	4.9384
229.4535	14.1081	231.2035	12.8164	230.2983	10.3377	233.0037	6.6571
236.1853	6.5845	238.3066	4.1733	235.9538	10.7965	238.7608	9.593
266.6344	0.59	263.3678	2.879	267.2103	0.4701	266.4567	4.1215
294.9203	10.0143	291.5486	37.4892	284.4329	1.1144	285.4142	2.6303
304.6391	91.5207	298.6507	78.0174	308.8124	64.6628	309.4062	54.7712
323.7358	31.3733	329.9246	11.0262	334.4075	19.1043	335.4425	20.3855
336.0275	127.3816	335.8121	124.9062	340.2411	10.8802	342.488	18.194
345.1463	2.0971	343.4198	8.426	348.4011	101.9793	355.207	89.1337
367.271	38.8522	365.847	26.1372	368.8557	77.2107	370.1156	72.7431
402.7656	5.4519	396.8602	10.73	392.1437	6.9952	392.3444	8.9422
439.6323	17.0144	440.1377	6.0288	398.4168	5.5685	417.9835	12.7545
445.287	5.6288	448.3326	38.7455	443.2915	31.0617	443.1317	12.9212
451.0076	28.8419	466.8408	2.3805	450.978	21.6644	449.8768	29.5418
509.6462	54.2031	509.3571	27.9843	504.5352	47.5302	504.8531	31.3226
531.4864	11.1451	530.1186	19.4527	517.8574	123.189	529.7189	19.5923
572.3114	8.6706	567.5325	1.2723	530.2456	20.0731	542.0848	122.6667
578.8892	23.3039	570.9427	11.8798	565.9641	7.3362	565.0572	2.8145
603.2731	28.2892	602.5359	6.2908	573.5233	30.3502	565.9791	15.1038
617.1722	10.6451	616.0855	11.6037	593.0729	53.5838	596.0329	17.5721
639.9266	34.0723	643.1725	9.6297	612.9697	2.3915	611.1058	1.6606
649.9213	1.0564	652.7447	1.2748	630.0486	55.4181	633.5691	30.4698
657.8046	15.4282	659.4567	16.9363	651.8308	14.2257	655.1253	16.0071
682.9435	61.8003	684.6782	49.7661	655.474	6.6415	656.0382	7.0345

700.5098	19.4389	703.7206	15.9673	682.2662	27.8823	684.3646	26.5003
746.217	81.2058	748.4798	83.3053	698.49	13.5575	701.437	12.62
758.6379	0.2058	760.7226	2.8504	742.9776	84.0332	743.9791	85.2587
774.9379	1.4769	774.2306	2.6293	759.4726	4.2354	762.1262	8.6301
776.7528	9.7721	778.9971	3.0604	769.5933	4.1403	770.8869	5.2394
804.2747	3.1276	791.6613	49.7457	774.605	6.5419	778.1542	7.3209
888.32	2.0295	833.0988	78.3045	802.634	1.7181	795.3873	43.0863
904.8124	25.2981	890.7303	1.7283	883.2496	0.3626	839.8419	88.7313
918.1497	29.7463	904.0498	49.0022	899.1949	20.2032	886.1894	0.5805
922.1427	58.8266	917.1546	2.3516	920.0494	21.3983	898.9247	21.6711
950.6582	6.8588	922.6392	75.5422	920.7074	15.0564	920.1995	1.8081
961.3417	44.1777	939.1686	12.2328	961.3886	29.5805	925.247	2.3051
967.2793	29.2403	965.9269	48.6379	968.5972	234.868	962.6128	1.6051
986.2025	89.2629	969.1393	48.3889	975.0446	10.1866	968.5242	275.4619
1014.401	43.1034	1000.1445	98.643	987.3595	95.7077	974.9656	26.8001
1024.6594	93.8827	1024.3538	97.8716	1027.49	75.7607	1003.5774	70.5938
1053.1595	63.4071	1041.2905	70.0524	1041.2276	13.1488	1042.9212	35.6656
1054.1614	1.8018	1051.0263	156.3956	1052.6172	24.2284	1046.7107	37.5108
1063.2631	4.825	1057.0042	8.161	1054.7065	2.1739	1051.9292	37.6266
1072.3795	22.3116	1062.2936	14.0916	1059.7115	70.8879	1056.6029	161.9838
1092.4203	6.451	1083.2808	40.3176	1071.5171	46.6968	1057.647	12.6454
1142.6155	128.6592	1092.3211	33.5676	1107.0523	37.0297	1078.5259	62.6373
1147.7305	243.7763	1143.7779	31.3348	1150.3327	64.9879	1106.7653	55.613
1188.5759	74.2579	1148.8052	602.6121	1177.9675	112.146	1151.1638	75.409
1201.2832	83.3902	1195.2566	216.8548	1198.7126	204.0855	1179.6571	165.9928
1214.6423	358.7143	1207.4584	14.3404	1206.0388	65.4278	1207.8442	13.7078
1243.6214	95.7354	1223.4853	274.5686	1216.6363	219.0362	1213.4541	111.871
1289.7671	167.5888	1249.93	180.3858	1273.1971	232.0783	1216.042	398.0919
1317.2424	30.8396	1268.1352	130.5681	1294.0709	166.5074	1262.4743	205.5438
1324.2006	88.5842	1323.8783	59.0003	1312.3569	58.4949	1276.2891	189.7643
1351.2932	108.1529	1332.1388	0.3203	1322.7238	165.2538	1315.8471	58.4398
1371.2773	19.9322	1360.5353	143.4564	1354.1753	18.3829	1337.6255	93.9221
1391.2807	126.7119	1376.3857	16.5321	1372.0199	37.2393	1357.2029	12.0195
1402.1303	28.6915	1392.4977	206.9807	1386.7593	29.0533	1380.0258	60.4457
1409.4445	7.2137	1404.021	29.6811	1405.2199	108.5636	1390.0239	76.7781
1411.4963	34.0322	1411.9363	51.3229	1408.4872	48.5181	1402.9291	80.1814
1415.5925	20.0962	1412.9354	76.6479	1411.6762	0.5137	1408.9716	64.6603
1457.4174	203.9215	1426.3261	18.5483	1417.0642	212.122	1414.9605	2.9473
1464.8787	21.3011	1437.4837	44.773	1420.7118	95.314	1420.6026	351.9999
1465.5558	17.0812	1451.6837	16.9555	1460.0788	17.9458	1425.9398	88.8914
1466.3335	10.8932	1458.9149	63.0801	1464.4117	14.7885	1436.2037	79.7805
1473.3893	11.1109	1463.6842	247.8076	1466.8189	11.691	1450.8618	18.6712
1485.1559	5.3282	1471.5012	263.5097	1476.7217	9.3599	1458.9756	18.1684
1513.5925	6.9834	1479.6504	570.6107	1482.1344	30.9037	1475.7851	6.0873
1526.8325	810.7104	1498.0003	444.1149	1508.9895	54.4579	1480.9688	13.645
1552.6771	59.2022	1512.8321	99.6803	1514.2641	188.6098	1505.0103	41.3921

1562.8265	159.2697	1528.5435	522.1949	1547.8893	106.9618	1512.5107	52.2985
1600.0027	6.0831	1561.0119	292.5913	1555.593	343.3316	1536.4532	414.7113
1621.045	418.3757	1598.3538	322.1479	1592.9315	337.7863	1544.7093	382.2326
1652.5525	266.8038	1621.0639	232.2423	1600.7668	337.2323	1595.1149	464.5868
3033.3798	32.4895	1649.3826	88.6882	1603.403	902.8543	1598.8473	1306.133
3035.0428	56.0856	1668.3012	79.0217	1669.8588	54.9952	1617.4629	0.8649
3035.2456	39.5586	3033.1292	18.07	3037.9033	15.2896	1660.8072	41.7198
3076.6354	28.2619	3047.8632	55.1597	3041.6972	60.2946	1683.8014	16.9218
		3049.5766	2.2052	3048.2471	2.3202	3047.987	1.8753
		3087.0641	13.7439	3080.7069	25.2325	3051.1781	2.0392
		3091.3105	17.4394	3084.7411	13.9297	3053.6252	39.2984
						3094.9212	16.614
						3100.1521	1.4811
						3104.8373	0.1515

Table S6. Calculated TD-DFT spectra of different protonated species of complex formed by gold(III) and hydrazone derived from pyridoxal and 3-furoylhydrazide (**PL-F3H**)

Deprotonated complex, [AuClL]		Monoprotonated complex 1, $[AuCl(HL)]^+$		Monoprotonated complex 2, $[AuCl(HL)]^+$		*Bis*-protonated complex, $[AuCl(H_2L)]^{2+}$	
λ, nm	oscillator strength	λ, nm	oscillator strength	λ, nm	oscillator strength	λ, nm	oscillator strength
473.47	0	441.18	0	519.32	0	450.11	0
369.16	0.0016	384.27	0.4225	374.6	0.0819	373.72	0.0769
365.97	0.2165	348.59	0	364.83	0.0001	366.51	0
315.86	0.0279	329.58	0.0048	349.85	0.0642	359.37	0.1719
315.06	0.0032	325.84	0.0669	333.88	0.0001	335.86	0.0001
301.06	0.5301	315.8	0.2582	309.19	0.0008	311.17	0.342
299.53	0.0006	305.48	0.0002	306.94	0.0118	300.97	0
290.38	0.0056	290.93	0.0479	299.46	0.1767	284.5	0.011
280.02	0.0364	283.71	0.0859	287.64	0.6095	276.6	0.0893
266.41	0.1803	270.9	0.0004	276.31	0.0286	260.56	0.0227
258.68	0.1662	259.16	0.161	270.5	0.002	254.92	0.3955
254.35	0.009	249.86	0.0104	263.8	0.1794	252.19	0.0372
244.12	0.0004	243.17	0.0001	251.21	0.0296	244.93	0.1672
238.56	0.1812	236.68	0.0722	233.03	0.0834	236.32	0.0148
230.8	0.1195	233.56	0.438	230.71	0.1179	231.46	0.1077
226.15	0.0404	231.23	0.0103	229.27	0.1069	229.24	0.0685
225.74	0.0571	227.48	0.0562	220.24	0.1539	224.62	0.1822
217.62	0.0223	223.83	0.1038	218.55	0.0052	219.1	0.0354
214.34	0.0081	219.66	0.0527	217.18	0.0892	218.84	0.0039
213.29	0.3443	217.8	0.0337	216.15	0.184	216.82	0.0628
209.04	0.1121	215.46	0.0616	210.97	0.0684	210.19	0.0457
207.64	0.0587	210.38	0.1261	210.03	0.0022	207.96	0.1009
207.25	0.1007	207.95	0.0867	209.55	0.0283	207.26	0.301
206.88	0.0112	207.46	0.1227	207.86	0.1247	203.71	0.1943
205.44	0.0198	204.59	0.032	203.62	0.0033	195.64	0.0063
203.56	0.0159	198.58	0.0603	201.05	0.0873	195.09	0.0016
202.35	0.0756	197.61	0.0346	200.79	0.046	194.06	0.0033
198.54	0.1019	194.39	0.2663	197.99	0.1186	192.86	0.0365
194.91	0.0033	193.02	0.0647	194.76	0.1343	189.83	0.0039
193.66	0.0746	189.29	0.0088	189.3	0.0825	187.72	0.1788

Table S7. Optimized geometry (*xyz*-coordinates) of different protonated species of complex formed by gold(III) and hydrazone derived from pyridoxal and thiophene-2-hydrazide (**PL-T2H**)

Deprotonated complex, [AuClL]

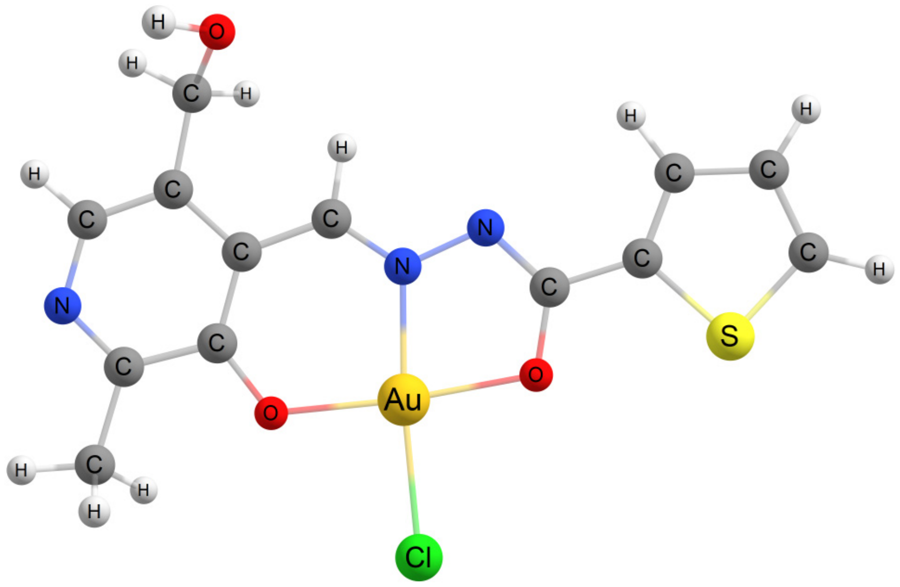

6	-4.130776000	-0.547229000	-0.034734000
6	-2.739875000	-0.204656000	-0.044022000
6	-2.384500000	1.163080000	-0.116197000
6	-3.435973000	2.121627000	-0.197891000
6	-4.732953000	1.669184000	-0.183792000
7	-5.072847000	0.369848000	-0.102409000
1	-5.552971000	2.374248000	-0.241982000
6	-4.532405000	-1.988380000	0.056132000
1	-4.136277000	-2.449926000	0.962317000
1	-4.135961000	-2.560158000	-0.784761000
1	-5.616706000	-2.060868000	0.060350000
8	-1.926794000	-1.245086000	0.018391000
6	-1.033102000	1.652054000	-0.113503000
1	-0.867467000	2.720464000	-0.125021000
6	-3.181665000	3.605623000	-0.312160000
1	-4.138084000	4.114655000	-0.445899000
1	-2.571657000	3.828053000	-1.188295000
8	-2.466092000	4.155789000	0.797920000
7	0.036138000	0.928683000	-0.071018000
7	1.280992000	1.498293000	-0.074445000
6	2.228373000	0.600194000	-0.036894000
8	2.014647000	-0.704031000	0.003347000
6	3.616137000	1.023315000	-0.039195000

6	4.116142000	2.303333000	-0.087209000
6	5.527789000	2.341109000	-0.073226000
1	3.482944000	3.175631000	-0.131082000
1	6.106832000	3.251353000	-0.105145000
16	4.900022000	-0.147784000	0.023897000
6	6.086931000	1.093311000	-0.014804000
79	0.042786000	-1.058871000	0.008995000
17	0.258426000	-3.379083000	0.097962000
1	7.131868000	0.833119000	0.007698000
1	-2.985021000	4.006665000	1.596295000

Monoprotonated complex, $[AuCl(HL)]^{+}$ 1, proton is bound with heterocyclic nitrogen of pyridoxal

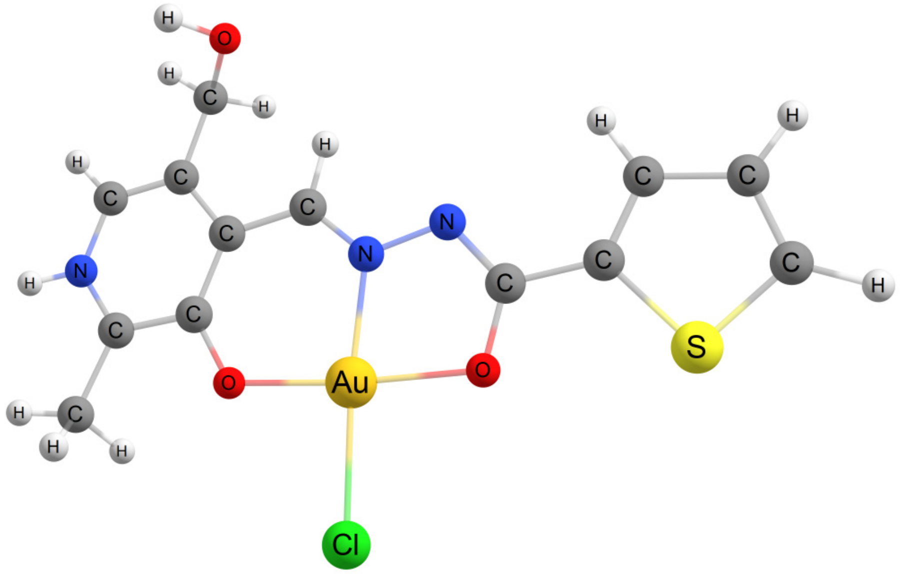

6	-4.083246000	-0.578593000	-0.045918000
6	-2.709594000	-0.215168000	-0.039778000
6	-2.364271000	1.160600000	-0.105137000
6	-3.406021000	2.127668000	-0.194412000
6	-4.707156000	1.709773000	-0.198086000
7	-4.991883000	0.390673000	-0.123703000
1	-5.549300000	2.378364000	-0.258470000
6	-4.520492000	-1.996305000	0.031512000
1	-4.142928000	-2.454102000	0.946128000
1	-4.103801000	-2.561306000	-0.802571000
1	-5.604384000	-2.076903000	0.012161000
8	-1.904440000	-1.249783000	0.028245000
6	-1.009375000	1.656072000	-0.096392000
1	-0.846197000	2.723924000	-0.101628000
6	-3.140329000	3.615931000	-0.300021000
1	-4.091824000	4.134857000	-0.421279000
1	-2.542788000	3.824825000	-1.187135000
8	-2.405973000	4.137497000	0.801122000

7	0.057721000	0.929385000	-0.058351000
7	1.291749000	1.493779000	-0.062440000
6	2.245638000	0.592316000	-0.031598000
8	2.027640000	-0.708933000	0.007076000
6	3.626304000	1.016990000	-0.041722000
6	4.115895000	2.303415000	-0.094078000
6	5.524800000	2.351166000	-0.090574000
1	3.475107000	3.170231000	-0.134041000
1	6.097930000	3.264723000	-0.127311000
16	4.919155000	-0.145073000	0.012290000
6	6.092623000	1.105559000	-0.035707000
79	0.067137000	-1.063339000	0.015850000
17	0.284920000	-3.373253000	0.096960000
1	7.139864000	0.853856000	-0.020864000
1	-5.968573000	0.118322000	-0.127049000
1	-2.946637000	4.069779000	1.596350000

Monoprotonated complex, $[AuCl(HL)]^{+}$ 2, proton is bound with hydrazide nitrogen

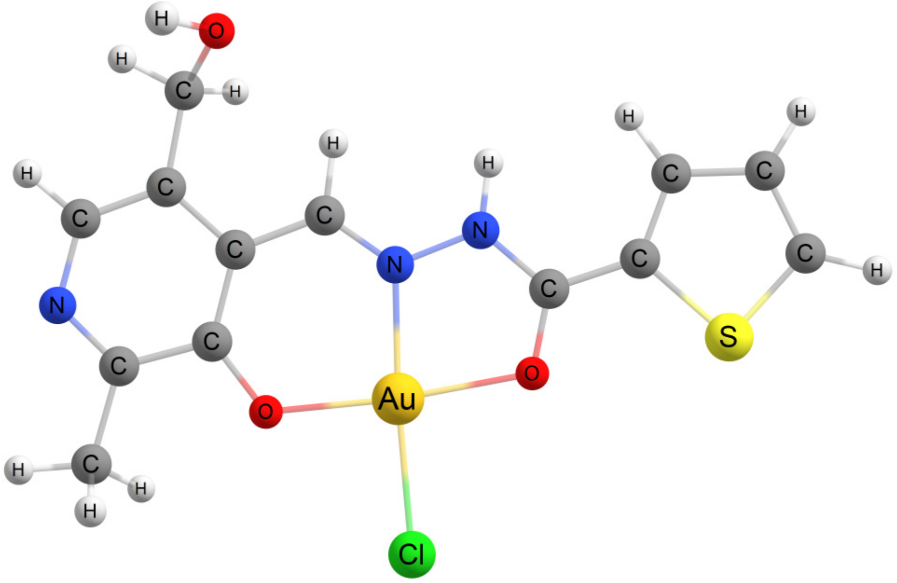

6	-4.134981000	-0.557091000	-0.042560000
6	-2.744176000	-0.200335000	-0.037744000
6	-2.403588000	1.172945000	-0.095863000
6	-3.461321000	2.126806000	-0.174266000
6	-4.751865000	1.661447000	-0.169528000
7	-5.076316000	0.356786000	-0.104299000
1	-5.579932000	2.356631000	-0.223412000
6	-4.525715000	-2.001041000	0.029339000
1	-4.138509000	-2.466622000	0.937141000
1	-4.112653000	-2.560256000	-0.811732000
1	-5.609127000	-2.080953000	0.017773000
8	-1.935086000	-1.244007000	0.027418000
6	-1.058740000	1.673115000	-0.082550000

1	-0.917438000	2.747200000	-0.066496000
6	-3.218823000	3.613846000	-0.293634000
1	-4.183376000	4.120575000	-0.348454000
1	-2.681795000	3.839844000	-1.215619000
8	-2.416346000	4.157791000	0.756591000
7	-0.007749000	0.927568000	-0.054863000
7	1.272176000	1.430573000	-0.041896000
6	2.286986000	0.551265000	-0.030613000
8	2.022507000	-0.705287000	-0.003048000
6	3.647171000	0.991815000	-0.045854000
6	4.152694000	2.274788000	-0.153725000
6	5.556000000	2.304528000	-0.134698000
1	3.546758000	3.162886000	-0.250569000
1	6.141080000	3.207469000	-0.208085000
16	4.927461000	-0.182248000	0.076587000
6	6.104910000	1.051828000	-0.013653000
79	0.024527000	-1.066162000	0.009155000
17	0.218497000	-3.358965000	0.075325000
1	7.149384000	0.790862000	0.027517000
1	-2.880739000	4.036998000	1.592651000
1	1.398191000	2.434549000	-0.048075000

Bis-protonated complex, $[AuCl(H_2L)]^{2+}$

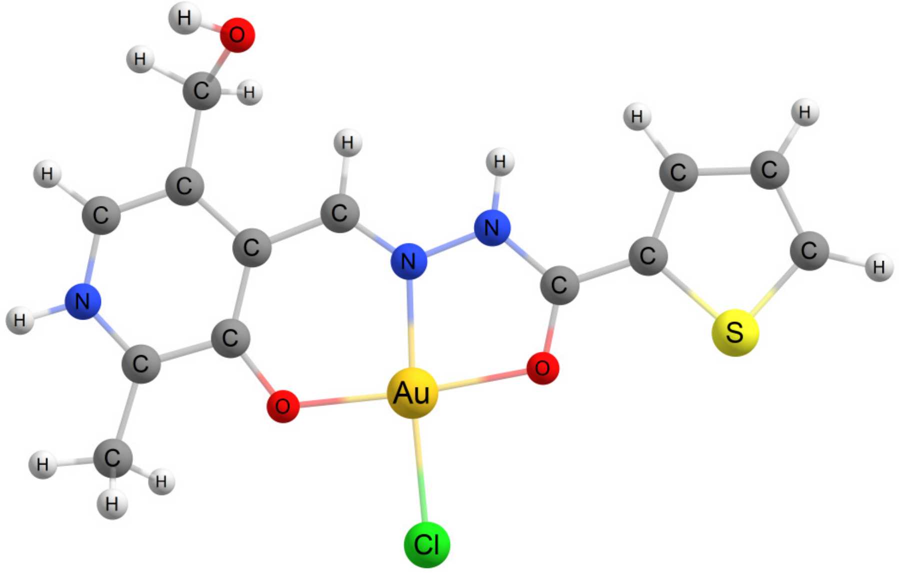

6	-3.897832000	-0.646713000	-0.035397000
6	-2.530256000	-0.243381000	-0.036304000
6	-2.228321000	1.140764000	-0.096741000
6	-3.293346000	2.083179000	-0.175998000
6	-4.581270000	1.627841000	-0.170037000
7	-4.826106000	0.299926000	-0.099676000
1	-5.443296000	2.271371000	-0.218270000
6	-4.294122000	-2.075335000	0.035404000

1	-3.894420000	-2.528132000	0.943072000
1	-3.868762000	-2.620835000	-0.807340000
1	-5.375164000	-2.185499000	0.025018000
8	-1.708846000	-1.266919000	0.022527000
6	-0.883982000	1.674396000	-0.089328000
1	-0.767947000	2.750827000	-0.077098000
6	-3.071702000	3.579468000	-0.295571000
1	-4.041923000	4.075395000	-0.319169000
1	-2.572381000	3.800098000	-1.239102000
8	-2.241264000	4.115622000	0.725410000
7	0.175121000	0.947659000	-0.060492000
7	1.440874000	1.467433000	-0.058142000
6	2.468002000	0.594452000	-0.031337000
8	2.225879000	-0.664627000	0.002015000
6	3.810282000	1.063942000	-0.039306000
6	4.383686000	2.315303000	-0.072932000
6	5.776648000	2.109550000	-0.062145000
1	3.876880000	3.264772000	-0.101079000
1	6.546025000	2.860275000	-0.080842000
8	4.785435000	0.106274000	-0.008746000
6	5.961227000	0.755496000	-0.023092000
79	0.248315000	-1.050517000	0.011717000
17	0.487839000	-3.328083000	0.101256000
1	6.841523000	0.137987000	-0.002639000
1	-2.703879000	4.064756000	1.569827000
1	1.556568000	2.473230000	-0.089710000
1	-5.796295000	0.002338000	-0.094415000

Table S8. Calculated IR spectra of different protonated species of complex formed by gold(III) and hydrazone derived from pyridoxal and thiophene-2-hydrazide (**PL-T2H**)

Deprotonated complex, [AuClL]		Monoprotonated complex 1, $[AuCl(HL)]^+$		Monoprotonated complex 2, $[AuCl(HL)]^+$		*Bis*-protonated complex, $[AuCl(H_2L)]^{2+}$	
Frequency, cm^{-1}	Relative intensity	Frequency, cm^{-1}	Relative intensity	Frequency, cm^{-1}	Relative intensity	Frequency, cm^{-1}	Relative intensity
29.8837	0.148	30.692	8.4732	28.2486	0.1554	30.0715	7.5698
31.788	0.4054	34.4848	0.1286	36.1288	2.1387	38.7229	2.8751
41.9649	0.3355	45.2688	0.0559	49.6232	3.0852	51.172	1.7395
62.4947	1.5172	63.0825	4.3309	63.5834	1.6821	65.0298	2.963
76.8139	1.9985	79.7675	4.6357	72.6222	2.7202	74.9681	3.0577
94.2964	1.304	96.6101	0.0326	92.7141	2.387	96.8891	0.0453
103.95	3.9864	103.2956	1.62	106.3702	0.4303	106.2142	0.5512
109.6324	2.0499	110.3369	4.4567	113.6098	5.0087	114.2052	5.4974
118.0949	0.7694	121.4484	0.2346	122.0362	6.0711	123.5069	1.9473
154.1496	0.1113	135.4712	0.6431	154.0779	0.6348	142.4672	1.1203
170.543	2.3721	170.8415	1.2277	168.7981	5.9381	170.6941	4.6959
176.1181	8.9257	176.8429	3.7691	174.6297	3.2928	176.6616	0.8266
187.5579	0.9479	184.7869	3.9257	183.7979	2.9039	185.4531	5.7646
200.3948	0.7494	203.6809	1.6216	194.9805	1.5364	198.1121	3.8017
211.6838	0.5578	210.2119	2.1649	209.834	3.021	211.0023	4.722
237.1238	8.1004	238.5311	5.6813	232.2619	12.7123	234.9892	8.6584
248.1629	4.2028	248.5627	2.3741	242.4302	5.5729	245.6015	4.9455
273.9874	1.1464	269.3317	4.404	271.0934	0.6083	270.3379	5.3729
307.734	133.5772	301.1291	128.315	314.9013	85.8626	314.7699	73.2165
324.7554	61.6873	332.4575	64.1073	339.9216	10.5802	344.6237	15.7923
343.0309	15.4315	340.868	19.8397	346.2592	52.2417	353.3799	47.9608
360.8568	89.7954	358.6784	69.2997	363.8673	130.2904	364.8384	117.3679
389.973	8.0139	390.7519	5.1714	384.1856	8.2609	387.251	5.4219
401.8464	9.7498	397.9503	18.5534	386.1539	0.4167	394.5168	10.8419
433.034	0.4111	442.5301	25.644	397.6753	8.6486	410.1176	14.2982
447.5062	35.9173	450.9673	0.4456	446.3545	40.3625	441.4772	28.3843
480.6081	3.7728	482.5542	12.1184	467.2698	0.6604	467.3335	0.8854
485.4465	37.0272	485.9611	18.4072	480.7173	47.8908	480.7699	39.3905
530.5975	13.446	528.0272	15.8096	509.8636	111.7359	528.5982	18.78
568.8135	3.8309	567.6248	5.8518	528.6617	22.1347	535.6759	104.3172
578.1434	16.7604	568.3399	1.6131	561.3435	3.7942	562.1675	8.04
581.4315	7.2808	579.4739	1.2756	572.1619	1.5261	566.8739	5.0512
586.1709	40.0578	585.2149	10.102	573.4403	44.7049	570.9836	22.0956
630.687	9.1336	630.4244	1.2446	577.2551	44.2795	578.6781	14.2842
655.6949	30.0123	658.0859	20.4455	622.7621	24.9715	621.9919	5.7339
681.6699	83.8967	683.3729	69.0291	649.6277	28.0387	654.5529	18.621
693.1166	13.5721	698.6055	23.2284	679.478	60.3504	681.2007	53.2397
726.4985	30.6503	730.055	44.3776	693.6096	35.7177	699.0102	48.6797
734.8441	100.2056	741.7535	86.6092	725.6115	36.025	726.1828	37.8092
752.9931	16.0786	754.0665	32.364	753.3097	19.9351	754.5425	27.5471

763.3861	10.5873	763.6513	10.9087	756.2226	81.2066	761.3953	81.5277
776.0357	8.0741	775.4305	2.7816	762.2569	45.6077	762.1847	47.6493
804.1126	4.3434	791.3629	53.8008	774.2316	6.7298	778.0302	14.3477
859.8662	82.0335	835.7042	81.3076	802.7928	3.036	796.0587	47.7854
879.7472	5.5895	861.9651	100.9907	865.9176	72.6625	844.1807	92.2915
903.8995	1.4935	884.105	4.043	882.9575	1.7191	868.5179	59.5065
918.404	29.6026	903.6501	2.0611	892.7407	12.9378	886.7066	1.4954
950.1984	0.3035	917.3229	1.8323	919.5571	29.1908	892.2819	25.4867
951.4405	7.3021	938.9485	11.8028	958.1559	0.2089	927.2593	2.8259
961.6988	17.5544	954.6384	0.6528	961.0693	4.1403	961.7419	0.1778
983.5856	65.7731	965.303	19.2868	973.9907	29.1474	963.2284	2.9001
1011.2946	116.3423	996.0897	68.557	985.3689	98.1152	976.9599	21.1746
1024.0904	111.4652	1019.6687	231.7825	1027.1503	86.3685	998.8159	64.1094
1054.0346	1.8422	1042.0355	117.8588	1038.525	26.9141	1041.0837	10.8987
1063.4049	15.2483	1057.6359	8.4848	1054.8218	1.7871	1046.5913	122.2294
1072.7545	6.4452	1065.0066	21.1154	1067.5814	32.6072	1058.3672	8.5891
1082.3359	28.9397	1081.1245	22.6422	1082.4912	16.6049	1070.6014	65.1019
1103.8971	3.4376	1085.8754	20.7974	1103.546	33.9174	1087.1704	45.6035
1146.3562	257.9673	1105.0964	2.9397	1115.587	172.3916	1104.9328	23.5683
1200.2018	71.5548	1156.7587	640.7275	1182.9744	163.3114	1117.513	247.7495
1212.6673	244.392	1206.6277	13.6793	1198.6379	259.6677	1186.0726	297.4851
1249.3596	56.4674	1222.421	216.3851	1205.842	69.657	1208.9425	17.6664
1289.5274	236.1169	1250.02	74.9166	1266.4388	12.8009	1214.8396	255.966
1310.3901	87.6522	1268.1138	213.7749	1292.8878	232.7294	1263.6085	163.2789
1323.9173	109.902	1317.3968	49.1085	1311.5684	62.8861	1266.7091	27.9993
1327.9397	209.5316	1328.9771	93.7417	1322.3042	198.9418	1318.3871	73.0285
1369.8928	31.1757	1344.1122	261.1865	1342.861	206.5439	1337.4103	112.1364
1391.0945	83.9637	1375.2053	19.5726	1371.8245	25.246	1346.9529	189.5871
1391.8454	84.1177	1389.8888	367.556	1375.5779	237.6366	1375.3798	122.3231
1407.8502	110.0157	1395.8574	44.4446	1391.2166	54.1857	1382.1101	242.6636
1411.295	68.8782	1411.4918	154.6119	1407.1117	110.7424	1390.849	261.8725
1421.0199	101.9135	1416.2171	225.6127	1411.4041	25.9512	1406.3366	63.2326
1451.8468	240.9234	1428.7158	9.1044	1415.8339	241.9733	1416.2893	294.4319
1464.8323	19.76	1438.0784	122.6834	1443.6746	443.0404	1421.3531	228.6371
1466.3164	11.1147	1447.1738	507.99	1464.1394	14.5438	1435.5669	41.7005
1484.8737	5.1096	1451.0911	17.5178	1466.5996	11.7892	1443.4063	479.0853
1512.6865	6.346	1477.4542	792.4226	1480.4718	316.6827	1450.1689	18.4062
1533.7265	974.2655	1496.7479	560.5992	1483.4352	121.1402	1477.0367	158.4127
1550.0727	112.297	1510.5649	122.785	1509.4949	14.8972	1488.1274	166.1712
1564.1064	116.8199	1526.4903	595.1237	1536.9148	81.2112	1506.3467	80.4309
1601.0527	58.544	1556.5057	232.2599	1559.7868	217.1718	1516.8393	82.8286
1649.6621	162.1484	1613.2509	22.7859	1595.9557	1330.5802	1547.6501	280.8747
3035.1108	36.0088	1646.9866	21.2556	1600.3703	34.915	1596.1371	1582.491
3035.4383	61.4659	1668.4354	62.8985	1667.4677	56.5898	1617.4528	6.6677
3077.0495	28.2459	3048.4547	57.4313	3037.8994	15.4063	1661.3193	40.5019
3080.8219	17.4629	3048.674	1.6567	3041.8429	61.3311	1682.9282	14.9284

3133.8387	23.6231	3091.8464	17.9491	3080.3306	25.9513	3050.2333	2.2305
3161.6748	47.5232	3103.2415	0.8014	3084.8145	13.7776	3053.8377	40.786
3198.0034	31.0732	3137.0052	18.2625	3138.6999	18.905	3094.3069	18.4417
3211.0724	7.7109	3208.7571	48.8637	3165.8421	73.107	3104.9223	0.131
3227.237	2.1677	3214.0773	6.3144	3169.3038	35.8265	3142.1352	14.5516
3248.61	2.1226	3229.5062	3.501	3213.659	0.5783	3170.2987	110.8041
3792.4541	67.7238	3249.1657	2.7603	3229.1411	3.4433	3215.6657	0.205
		3249.8984	2.6926	3250.9748	6.3596	3231.1892	3.7535
		3533.452	407.5653	3563.8923	159.5991	3251.0349	7.3904
		3792.8468	89.9772	3791.5955	76.8358	3251.0948	7.134
						3522.6155	358.0048
						3555.502	174.826
						3790.152	99.1826

Table S9. Calculated TD-DFT spectra of different protonated species of complex formed by gold(III) and hydrazone derived from pyridoxal and thiophene-2-hydrazide (**PL-T2H**)

Deprotonated complex, [AuClL]		Monoprotonated complex 1, $[AuCl(HL)]^+$		Monoprotonated complex 2, $[AuCl(HL)]^+$		*Bis*-protonated complex, $[AuCl(H_2L)]^{2+}$	
λ, nm	oscillator strength	λ, nm	oscillator strength	λ, nm	oscillator strength	λ, nm	oscillator strength
477.63	0	444.48	0.0002	521.72	0.0001	451.07	0.0001
371.3	0.2387	389.88	0.5408	377.64	0.1172	375.33	0.1118
370.64	0.0724	348.32	0.0001	366.24	0.0003	364.77	0.0003
317.13	0.0292	327.59	0.0805	351.66	0.0801	361.32	0.2194
310.25	0.0022	324.87	0.0007	322.44	0.0008	315.19	0.0025
308.01	0.5862	315.6	0.342	308.89	0.0952	307.83	0.6715
291.64	0.0033	291.77	0.0001	304.3	0.43	301.26	0.0003
290.07	0.0002	284.09	0.0434	301.55	0.0094	285.08	0.0053
279.85	0.0395	277.83	0.0331	292.24	0.5032	284.82	0.0455
266.41	0.376	269.89	0.0012	278.07	0.0122	276.74	0.0005
258.24	0.0673	265.36	0.015	270.4	0.0944	270.47	0.1391
254.36	0.0003	264.58	0.1048	266.33	0.001	260.9	0.0613
248.94	0.1509	259.66	0.1647	263.39	0.034	253.11	0.1385
247.89	0.0016	250.01	0.0092	259.16	0.1201	245.86	0.1481
244.4	0.0015	244.48	0.2417	251.67	0.0309	242.15	0.0085
237.3	0.0238	236.37	0.0092	239.43	0.0224	236.9	0.0073
232.74	0.0138	230.92	0.0898	233.09	0.0896	229.18	0.0474
228.01	0.0649	230.17	0.043	223.72	0.0321	227.56	0.0017
223.2	0.1162	228.18	0.0032	220.68	0.0149	222.43	0.0431
217.2	0.0402	221.35	0.0309	218.75	0.0047	219.82	0.0548
214.82	0.0305	218.35	0.0214	213.97	0.1198	218.4	0.0186
211.44	0.0089	215.92	0.0433	212.27	0.1159	213.04	0.1327
209.76	0.0541	211.13	0.1441	210.56	0.0039	210.07	0.2723
208.01	0.2139	209.15	0.066	209.31	0.0598	208.38	0.0377
207.64	0.0162	207.96	0.1865	206.59	0.0806	204.66	0.2196
207.25	0.009	206.96	0.021	205.53	0.0039	198.32	0.0014
204.6	0.0176	200.02	0.0186	204.52	0.0034	195.86	0.0144
202.18	0.0733	198.18	0.0804	202.22	0.0965	195.28	0.0344
200.17	0.0791	195.54	0.1648	199.57	0.1724	193.67	0.1023
196.02	0.0019	193.65	0.152	197.82	0.031	192.4	0.1014

Table S10. Optimized geometry (*xyz*-coordinates) of different protonated species of complex formed by gold(III) and hydrazone derived from pyridoxal and thiophene-3-hydrazide (**PL-T3H**)

Deprotonated complex, [AuClL]

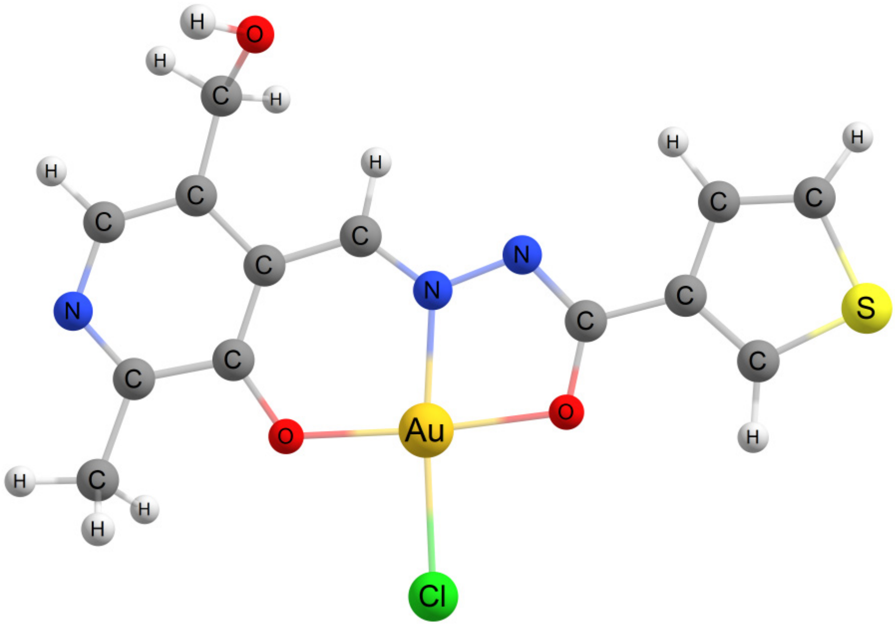

6	-4.190795000	-0.390403000	-0.028338000
6	-2.783832000	-0.118908000	-0.042559000
6	-2.360772000	1.229604000	-0.116265000
6	-3.362693000	2.239939000	-0.198933000
6	-4.680618000	1.853528000	-0.181861000
7	-5.085265000	0.572875000	-0.095829000
1	-5.464360000	2.598609000	-0.241121000
6	-4.664093000	-1.809316000	0.067352000
1	-4.286839000	-2.288585000	0.972396000
1	-4.301284000	-2.401721000	-0.774441000
1	-5.750607000	-1.827537000	0.077057000
8	-2.023986000	-1.198187000	0.015449000
6	-0.986708000	1.650140000	-0.113340000
1	-0.767793000	2.708964000	-0.127162000
6	-3.033486000	3.708785000	-0.317957000
1	-3.962810000	4.265306000	-0.453262000
1	-2.413097000	3.896779000	-1.194879000
8	-2.290383000	4.225588000	0.790065000
7	0.044512000	0.874329000	-0.067395000
7	1.318078000	1.382023000	-0.069966000
6	2.219578000	0.439141000	-0.031966000
8	1.939439000	-0.851377000	0.006537000

6	3.634470000	0.801845000	-0.032090000
6	4.125981000	2.144938000	-0.090893000
6	5.482346000	2.203785000	-0.077393000
1	3.485487000	3.010781000	-0.141353000
1	6.114031000	3.074807000	-0.112506000
6	4.646163000	-0.124472000	0.024892000
1	4.555481000	-1.195162000	0.076163000
16	6.186454000	0.624337000	0.007877000
79	-0.045704000	-1.109766000	0.008708000
17	0.060005000	-3.438290000	0.087538000
1	-2.816033000	4.106354000	1.589054000

Monoprotonated complex, $[AuCl(HL)]^{+}$ 1, proton is bound with heterocyclic nitrogen of pyridoxal

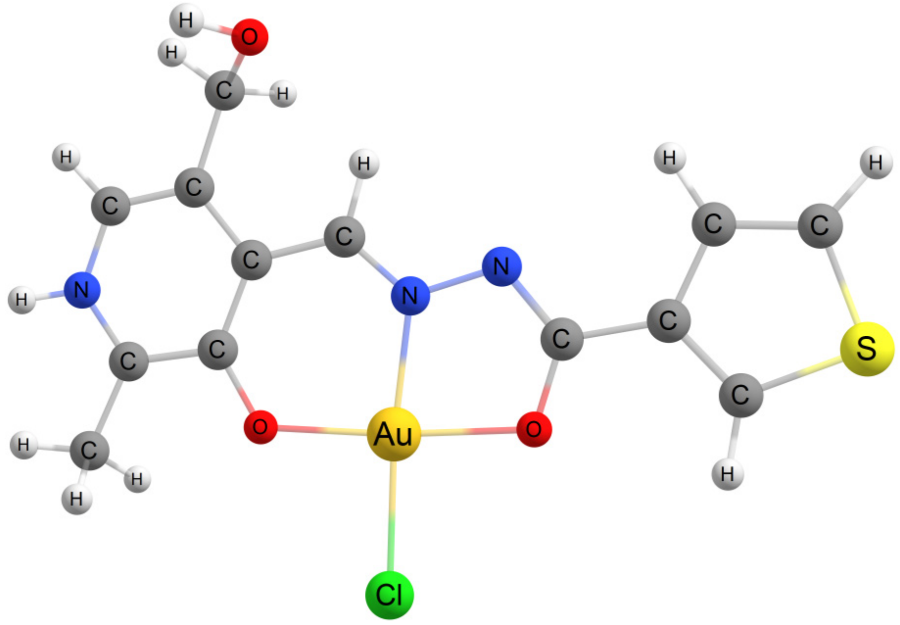

6	-4.144649000	-0.429973000	-0.036582000
6	-2.753765000	-0.134627000	-0.035393000
6	-2.343109000	1.222986000	-0.103717000
6	-3.335766000	2.239645000	-0.198707000
6	-4.655654000	1.885910000	-0.201512000
7	-5.003892000	0.582279000	-0.119062000
1	-5.464748000	2.593736000	-0.268002000
6	-4.651919000	-1.823512000	0.051040000
1	-4.291698000	-2.294612000	0.965904000
1	-4.269291000	-2.413070000	-0.782419000
1	-5.738794000	-1.849803000	0.039009000
8	-1.999854000	-1.206158000	0.029857000
6	-0.965440000	1.652597000	-0.092542000
1	-0.751194000	2.711512000	-0.099188000
6	-2.996728000	3.712509000	-0.312250000
1	-3.921135000	4.277007000	-0.438956000

1	-2.387249000	3.885985000	-1.198875000
8	-2.240007000	4.203597000	0.787731000
7	0.064147000	0.875566000	-0.050652000
7	1.326553000	1.381561000	-0.052711000
6	2.235332000	0.436908000	-0.024677000
8	1.954127000	-0.851087000	0.008490000
6	3.644031000	0.803408000	-0.032505000
6	4.129205000	2.149309000	-0.090085000
6	5.484841000	2.211525000	-0.086898000
1	3.485345000	3.012954000	-0.132554000
1	6.114245000	3.084056000	-0.123785000
6	4.660462000	-0.120945000	0.013186000
1	4.573673000	-1.192197000	0.060862000
16	6.194289000	0.633142000	-0.012917000
79	-0.019384000	-1.114032000	0.015815000
17	0.093505000	-3.432170000	0.077977000
1	-5.992744000	0.357506000	-0.119571000
1	-2.786109000	4.170990000	1.581458000

Monoprotonated complex, $[AuCl(HL)]^{+}$ 2, proton is bound with hydrazide nitrogen

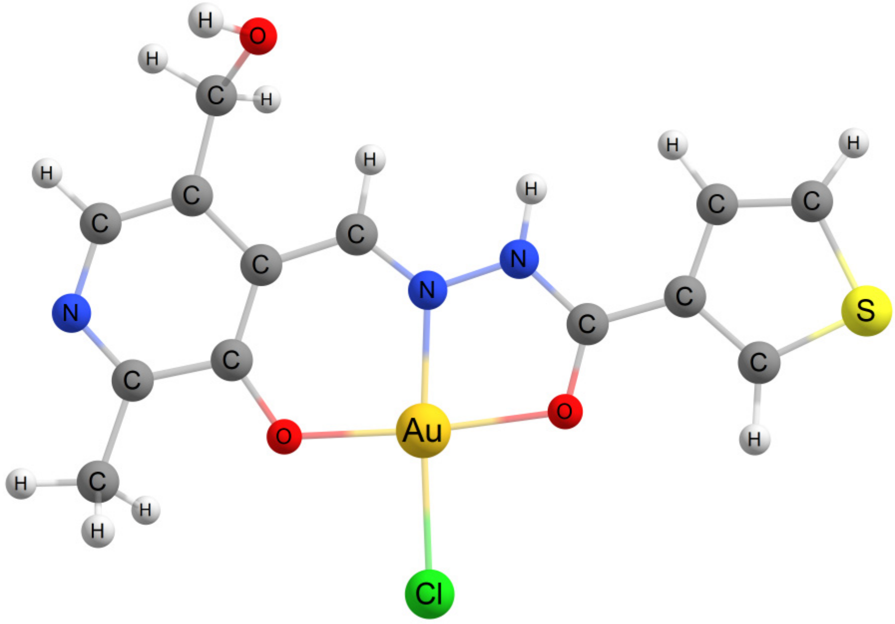

6	-4.194042000	-0.407366000	-0.026163000
6	-2.787271000	-0.117841000	-0.031084000
6	-2.382338000	1.238252000	-0.093199000
6	-3.393918000	2.241436000	-0.170727000
6	-4.704870000	1.838081000	-0.158243000
7	-5.090614000	0.550295000	-0.085703000
1	-5.499440000	2.571364000	-0.211499000
6	-4.652854000	-1.830724000	0.053415000
1	-4.280756000	-2.311649000	0.959535000
1	-4.274009000	-2.411379000	-0.789233000

1	-5.738887000	-1.858935000	0.050837000
8	-2.028887000	-1.198734000	0.027256000
6	-1.015663000	1.673300000	-0.082775000
1	-0.820723000	2.739313000	-0.070559000
6	-3.081097000	3.714584000	-0.300723000
1	-4.020464000	4.267185000	-0.347824000
1	-2.544435000	3.908755000	-1.230201000
8	-2.241071000	4.225422000	0.736589000
7	-0.002388000	0.877178000	-0.051296000
7	1.300641000	1.318069000	-0.037165000
6	2.271620000	0.394676000	-0.027699000
8	1.950331000	-0.845918000	-0.007922000
6	3.659903000	0.787120000	-0.038465000
6	4.164738000	2.120159000	-0.186976000
6	5.519657000	2.159196000	-0.155658000
1	3.561625000	3.004426000	-0.323359000
1	6.162727000	3.017267000	-0.247754000
6	4.665567000	-0.147042000	0.099501000
1	4.557984000	-1.209769000	0.229123000
16	6.200567000	0.581608000	0.055521000
79	-0.061978000	-1.114876000	0.006093000
17	0.029338000	-3.414458000	0.055460000
1	1.473873000	2.314844000	-0.025370000
1	-2.702073000	4.135890000	1.578393000

Bis-protonated complex, $[AuCl(H_2L)]^{2+}$

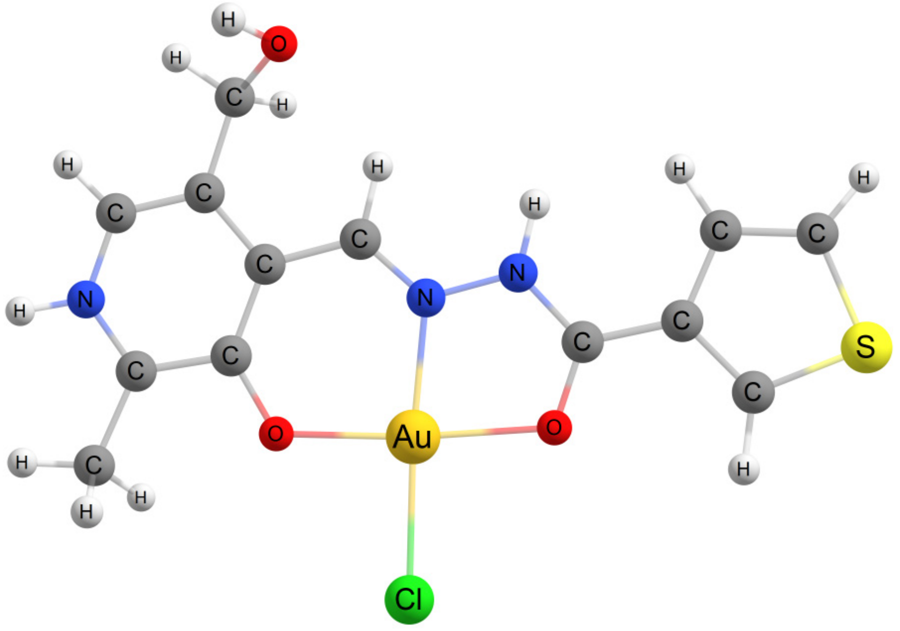

6	-4.148588000	-0.455416000	-0.042058000
6	-2.758097000	-0.136672000	-0.026041000
6	-2.371947000	1.227409000	-0.079140000
6	-3.376301000	2.233800000	-0.165635000

6	-4.689286000	1.857475000	-0.177697000
7	-5.015609000	0.546425000	-0.116154000
1	-5.510557000	2.551738000	-0.236316000
6	-4.633870000	-1.856525000	0.023981000
1	-4.277300000	-2.330766000	0.938739000
1	-4.230012000	-2.429665000	-0.810946000
1	-5.719618000	-1.899225000	-0.002777000
8	-2.001496000	-1.207830000	0.037852000
6	-0.997284000	1.676615000	-0.061100000
1	-0.813089000	2.744023000	-0.040565000
6	-3.064160000	3.714577000	-0.283305000
1	-4.002118000	4.269219000	-0.293320000
1	-2.565002000	3.905716000	-1.233438000
8	-2.189354000	4.198206000	0.726513000
7	0.013446000	0.884339000	-0.034395000
7	1.310292000	1.325185000	-0.020483000
6	2.285812000	0.399360000	-0.020880000
8	1.961799000	-0.841675000	-0.003894000
6	3.668779000	0.790466000	-0.040234000
6	4.172506000	2.125825000	-0.179597000
6	5.526880000	2.162818000	-0.161834000
1	3.569054000	3.012110000	-0.299662000
1	6.170259000	3.020961000	-0.250857000
6	4.677263000	-0.146798000	0.077208000
1	4.570641000	-1.210960000	0.196112000
16	6.208761000	0.581469000	0.025353000
79	-0.033415000	-1.113102000	0.014680000
17	0.070321000	-3.403098000	0.056266000
1	-6.002486000	0.308749000	-0.125992000
1	1.484339000	2.322501000	-0.007542000
1	-2.644688000	4.180989000	1.576139000

Table S11. Calculated IR spectra of different protonated species of complex formed by gold(III) and hydrazone derived from pyridoxal and thiophene-3-hydrazide (**PL-T3H**)

Deprotonated complex, [AuClL]		Monoprotonated complex 1, $[AuCl(HL)]^+$		Monoprotonated complex 2, $[AuCl(HL)]^+$		*Bis*-protonated complex, $[AuCl(H_2L)]^{2+}$	
Frequency, cm^{-1}	Relative intensity	Frequency, cm^{-1}	Relative intensity	Frequency, cm^{-1}	Relative intensity	Frequency, cm^{-1}	Relative intensity
27.6172	0.1024	27.4851	7.7841	26.906	0.0336	28.9272	6.3629
35.0597	0.1788	36.5543	0.4114	38.329	1.3361	41.1089	3.589
48.9454	0.6654	54.1988	0.1442	55.6767	4.3786	56.848	3.5055
60.5317	1.4078	61.0897	3.7513	63.6362	0.8679	70.2527	1.6542
75.4251	2.0134	77.3742	3.9464	73.7461	4.0693	75.8476	3.9231
92.516	1.1739	94.5617	0.2856	88.2425	1.8251	91.0237	0.2042
105.1777	3.3518	106.2105	1.1462	109.6334	0.6755	111.3252	0.8158
111.2205	1.582	111.7637	3.837	113.7028	3.8251	116.7229	4.8022
118.5923	1.4305	123.9787	0.8464	122.4019	9.2966	129.2421	4.715
159.0936	0.1506	148.0932	0.5434	156.6095	0.6485	145.6025	1.536
174.7125	7.8118	174.4441	2.252	168.7264	3.3522	171.5479	1.9689
183.5342	1.8168	183.0744	3.6852	182.1978	5.7068	182.475	3.0605
190.2963	0.8095	189.0582	2.0991	184.3383	2.5428	186.434	7.0814
200.7359	0.7417	205.3032	2.1482	193.2473	0.9135	196.3496	4.6872
210.711	1.0173	209.9624	3.1279	205.7252	4.3104	206.9348	5.8245
236.5845	4.7971	237.3592	1.9688	232.5789	11.029	234.1074	6.8213
251.8455	3.8131	252.6089	1.703	248.7063	5.134	251.0784	3.7034
277.876	1.7073	274.3013	5.1657	273.0561	0.7744	272.1755	4.4859
307.0467	134.8514	307.5508	114.1462	318.9658	61.5689	322.173	39.5771
324.9411	71.94	333.483	74.25	335.9558	11.7558	340.3046	12.4446
343.0572	14.2408	341.6732	17.3355	347.9	59.5419	356.0424	57.2468
361.8006	72.4973	362.1342	71.4869	365.6966	142.1114	371.1443	143.9932
401.1006	3.2789	395.6307	7.3871	384.9019	7.6666	387.672	7.9362
411.4084	17.8465	413.0048	17.163	393.8681	5.7461	407.8388	12.0488
440.1089	1.1306	445.565	23.7884	408.3423	6.6958	411.7586	5.6473
447.5675	33.7629	458.8861	2.1204	446.7178	39.7613	444.5187	28.5035
469.0113	0.4129	471.528	0.2544	466.6675	0.6033	467.0831	0.4186
485.7006	28.7124	485.5579	19.5812	480.2208	39.2739	481.0579	31.7966
530.6934	12.3619	529.2076	14.5421	503.7035	103.2496	524.6466	112.6153
572.3189	11.5035	569.1932	1.6879	530.9338	18.9756	531.6476	18.4046
578.2613	21.1341	570.9069	10.2936	567.6652	7.4964	566.0746	6.2392
596.8078	30.0503	595.0738	11.2341	572.3475	34.2293	568.9416	14.5878
604.8485	6.0222	604.2046	3.4872	589.1058	49.2968	589.1816	22.2748
639.7546	0.4207	639.4441	0.5313	599.1311	20.3529	597.6528	13.329
649.0613	73.4963	655.6883	29.4188	636.0398	81.4645	637.974	12.2048
660.9028	9.8474	664.8547	20.7282	640.3558	5.2	647.3957	34.6581
684.9004	36.3857	686.3239	30.5823	652.4459	10.4931	656.3677	19.633
713.7437	28.9107	716.4902	32.7423	683.2936	11.6767	684.0284	9.4359
735.2849	74.3431	738.8468	72.7652	716.1179	44.869	717.8768	47.8824
757.7908	0.7352	759.6665	3.3328	738.0043	49.4252	740.1186	47.4861

775.7971	7.4481	776.2129	1.5114	756.9245	2.3364	758.2718	5.4183
800.5819	13.9517	790.1441	56.6989	773.9753	6.1789	777.7812	10.783
805.7196	3.7725	802.4689	24.5695	798.4851	25.3986	793.7099	59.826
836.3404	45.6745	835.8798	63.2752	804.6152	5.5925	802.5677	19.0504
862.6486	160.9723	839.4276	56.6833	844.5656	43.9592	845.2562	99.7425
894.7256	6.5887	865.3876	202.8294	868.1595	107.408	846.7735	36.2469
918.4351	30.4123	896.7227	10.6503	902.513	54.3313	870.0365	91.2273
925.7547	0.552	920.9493	1.4698	914.6995	0.0604	905.6054	78.3694
952.5042	6.6369	927.1664	0.4505	919.4076	28.636	916.303	0.0183
957.8022	16.4762	940.9143	11.7258	957.2633	7.3061	929.2535	2.0525
968.2808	18.6367	962.4192	12.3923	964.3581	66.6005	955.1999	4.0932
991.4419	88.0561	969.8266	14.5019	974.7423	50.3682	967.4169	64.0631
1019.1274	39.7875	1001.5905	100.3826	990.6492	58.4462	975.6709	52.8063
1030.1959	190.4458	1032.1395	172.5435	1027.6195	78.5583	1003.1333	53.6588
1054.0947	1.7983	1045.8513	205.2627	1045.198	59.156	1043.4895	45.0014
1071.0267	18.5269	1057.983	9.066	1054.8153	1.7934	1053.653	173.4122
1088.5304	22.4557	1080.2434	34.0537	1070.0178	42.0429	1059.5695	10.1104
1102.6086	6.5675	1089.0199	78.6173	1119.728	2.6011	1076.4436	53.3829
1142.6982	111.3095	1105.0863	8.1401	1133.405	44.4736	1120.7712	2.1193
1199.3056	69.4709	1150.9353	237.7233	1177.4556	27.0413	1134.7908	54.0082
1210.5636	148.6633	1206.017	14.5398	1198.4115	268.3529	1180.5996	89.0181
1225.527	90.209	1221.5948	124.1537	1205.6345	49.2784	1210.1657	15.9333
1289.6651	215.7784	1229.2997	95.2692	1250.4621	23.2274	1214.0918	248.7736
1310.0934	154.468	1270.5585	145.3096	1292.8864	188.9153	1252.5765	21.518
1323.2609	130.92	1317.935	208.2056	1311.0457	66.1012	1268.0149	154.012
1325.6362	138.9546	1329.4746	110.9396	1321.5142	51.0302	1319.1178	134.25
1369.2489	20.1413	1341.2187	239.5857	1325.1105	417.1881	1325.8386	180.1276
1391.4861	128.9124	1375.1671	21.2581	1371.4546	41.7602	1338.1655	252.9993
1400.9341	43.9005	1392.3893	192.8216	1382.5653	0.118	1380.6886	112.2017
1410.028	7.7675	1402.6763	49.7287	1397.6861	194.1218	1384.7673	164.2247
1413.1045	44.0375	1412.0785	105.6573	1404.184	104.1255	1395.6289	86.8411
1425.9655	54.4707	1422.7514	147.7344	1411.6554	2.9929	1404.5927	99.8777
1459.229	130.1885	1426.9616	16.9933	1413.5296	169.9631	1414.3337	248.8308
1465.0039	24.5185	1437.9994	56.2576	1454.7099	232.1541	1422.3839	13.8231
1466.689	13.2477	1452.7957	16.9058	1464.5709	8.6344	1436.494	50.7515
1485.0262	3.2614	1460.3117	135.6936	1466.8124	11.8927	1450.927	18.3375
1511.6825	6.6445	1481.7738	475.7961	1482.7003	51.3212	1455.5042	335.175
1530.9058	585.6924	1501.8561	580.139	1501.5179	313.702	1482.762	44.0536
1552.6116	25.6853	1510.0008	119.2185	1508.6609	57.3089	1497.6395	120.994
1568.6721	518.571	1530.7184	572.8872	1548.6327	152.7538	1508.7019	119.3267
1601.7883	77.2276	1554.9854	535.7885	1551.2829	266.9192	1527.8841	525.5469
1650.5532	178.6465	1616.804	43.8987	1590.9777	1148.3749	1548.2386	48.5887
3035.237	25.9053	1650.7512	32.9038	1600.8561	41.79	1588.6901	1461.8212
3036.0841	70.6858	1669.868	65.242	1667.0673	69.174	1619.4354	5.8585
3076.992	29.0597	3048.6317	2.7015	3038.0233	15.0361	1663.7122	45.5725
3080.8667	17.3148	3049.9963	52.3205	3041.9497	60.5284	1683.6189	19.6216

3134.3226	23.2023	3091.8821	19.0801	3079.8025	27.4499	3049.53	2.093
3162.4752	45.6699	3103.1246	0.7331	3084.945	13.5508	3054.6969	35.6572
3196.3643	30.0781	3137.3588	15.6893	3139.0651	18.8372	3094.6857	21.5102
3226.802	2.1991	3206.428	48.1634	3169.3365	81.4804	3104.8134	0.1032
3253.5174	0.6998	3228.5918	3.2214	3170.184	34.2652	3140.3879	13.2417
3261.3515	7.5785	3251.6453	3.923	3215.7677	0.569	3171.4866	129.8298
3792.0461	68.2048	3254.9209	1.4073	3259.0008	9.159	3217.3362	0.6582
		3261.5316	8.9888	3262.4685	14.2639	3257.5095	9.5576
		3542.7736	392.3161	3573.6199	164.7467	3259.8369	13.4429
		3791.1307	91.068	3792.4833	76.112	3262.378	13.7978
						3526.3431	355.5317
						3565.5619	179.8241
						3793.1983	97.7874

Table S12. Calculated TD-DFT spectra of different protonated species of complex formed by gold(III) and hydrazone derived from pyridoxal and thiophene-3-hydrazide (**PL-T3H**)

Deprotonated complex, [AuClL]		Monoprotonated complex 1, $[AuCl(HL)]^{+}$		Monoprotonated complex 2, $[AuCl(HL)]^{+}$		*Bis*-protonated complex, $[AuCl(H_2L)]^{2+}$	
λ, nm	oscillator strength	λ, nm	oscillator strength	λ, nm	oscillator strength	λ, nm	oscillator strength
467.88	0	432.77	0.0002	516.73	0	446.71	0.0001
368.43	0.0081	380.53	0.4259	377.08	0.0906	374.51	0.0659
366.64	0.2199	348.15	0	365.5	0.0002	363.59	0.0007
316.14	0.0275	326.27	0.0582	351.09	0.0639	359.82	0.1824
306.05	0.0002	319.98	0.0006	316.36	0.0006	311.72	0.002
300.26	0.5547	309.84	0.4231	309.39	0.0106	301.5	0.0005
291.93	0.0115	292.02	0.0001	302.38	0.0004	297.69	0.4448
288.18	0	284.95	0.0205	298.87	0.0996	286.6	0.1033
279.16	0.0333	282.4	0.0296	284.03	0.7723	284.42	0.1359
262.49	0.4246	270.44	0.0005	275.41	0.1674	278.34	0.0001
256.88	0.0037	264.91	0.0005	271.49	0.0002	266.94	0.2365
254.3	0.0027	260.48	0.1655	268.87	0.1474	261.08	0.0979
244.77	0.0624	256.72	0.1506	258.03	0.0273	250.51	0.15
244.27	0.2224	249.89	0.0129	251.53	0.1308	241.37	0.0095
242.94	0.0625	237.21	0.0042	250.76	0.0773	237.72	0.0044
235.04	0.0038	232.61	0.2809	233.82	0.0299	237.19	0.0927
227.93	0.002	230.54	0.0512	228.3	0.0578	230.39	0.031
225.9	0.0944	226.61	0.0024	223.17	0.033	225.32	0.0625
218.71	0.0319	225.55	0.1168	218.77	0.0051	222.27	0.0553
215.17	0.0233	220.14	0.0675	216.12	0.0733	219.12	0.0678
213.02	0.2074	217.22	0.0419	213.2	0.1016	217.4	0.1339
210.51	0.0121	215.71	0.092	211.89	0.1486	210.9	0.0355
209.57	0.1409	210.71	0.252	210.45	0.0074	208.89	0.3313
207.5	0.0142	208.05	0.0669	208.96	0.0893	208.55	0.0497
207	0.0722	206.99	0.0408	205.77	0.0573	204.35	0.2635
206.71	0.1414	206.54	0.046	204.49	0.0084	196.81	0.0018
204.86	0.0051	199.1	0.0396	204.28	0.0245	195.97	0.0067
204.15	0.0304	197.89	0.1199	201.88	0.1193	195.11	0.0277
200.05	0.1213	192.71	0.1571	199.07	0.1621	193.24	0.2282
195.9	0.0023	192.58	0.0239	194.84	0.1081	190.63	0.0108

Table S13. Optimized geometry (*xyz*-coordinates) of different protonated species of complex formed by gold(III) and hydrazone derived from pyridoxal and isoniazid (**PL-INH**)

Deprotonated complex, [AuClL]

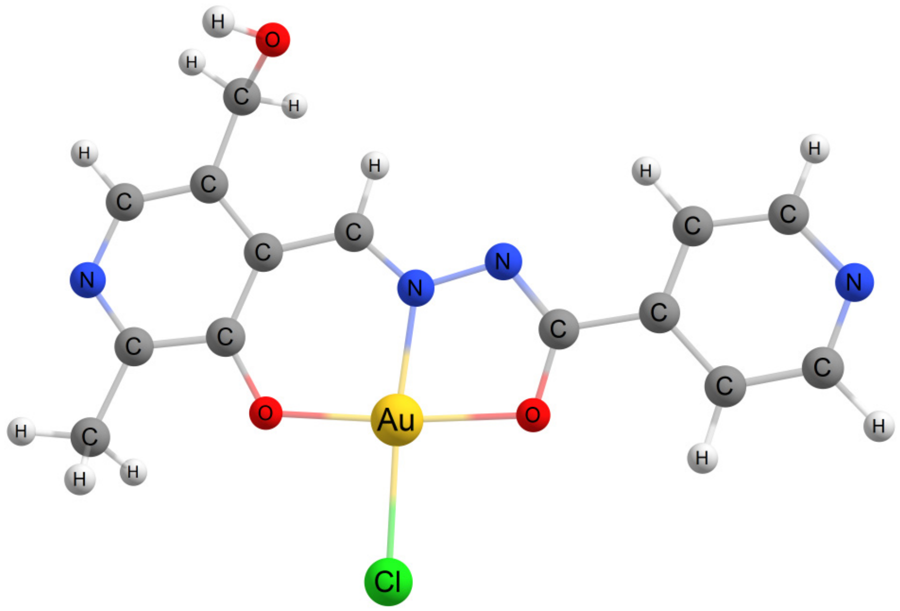

6	-4.138275000	-0.359589000	-0.028430000
6	-2.727951000	-0.100925000	-0.043617000
6	-2.292923000	1.244433000	-0.114181000
6	-3.285677000	2.264982000	-0.192321000
6	-4.606343000	1.890433000	-0.174325000
7	-5.022256000	0.612521000	-0.091760000
1	-5.383832000	2.642189000	-0.229769000
6	-4.624561000	-1.774150000	0.063380000
1	-4.251577000	-2.259126000	0.967129000
1	-4.267210000	-2.367361000	-0.780132000
1	-5.711147000	-1.782153000	0.073170000
8	-1.979712000	-1.187471000	0.010069000
6	-0.916216000	1.651190000	-0.111689000
1	-0.685918000	2.707716000	-0.124306000
6	-2.942310000	3.730975000	-0.308217000
1	-3.866986000	4.297503000	-0.432789000
1	-2.328271000	3.915626000	-1.190322000
8	-2.183413000	4.235078000	0.794597000
7	0.105784000	0.862816000	-0.066930000
7	1.385443000	1.357921000	-0.068950000
6	2.273566000	0.408232000	-0.030789000
8	1.987073000	-0.876952000	0.006687000

79	-0.002205000	-1.120212000	0.006414000
17	0.080538000	-3.447922000	0.081984000
6	3.710047000	0.760966000	-0.028348000
6	4.131608000	2.088485000	-0.109116000
6	5.492132000	2.354653000	-0.102783000
7	6.432442000	1.407693000	-0.022228000
6	6.019290000	0.140951000	0.055148000
6	4.682199000	-0.233652000	0.055149000
1	3.415614000	2.892810000	-0.176341000
1	5.842183000	3.377934000	-0.165799000
1	6.791454000	-0.616147000	0.120432000
1	4.406158000	-1.274623000	0.119671000
1	-2.704441000	4.123260000	1.597685000

Monoprotonated complex, $[AuCl(HL)]^{+}$ 1, proton is bound with heterocyclic nitrogen of pyridoxal

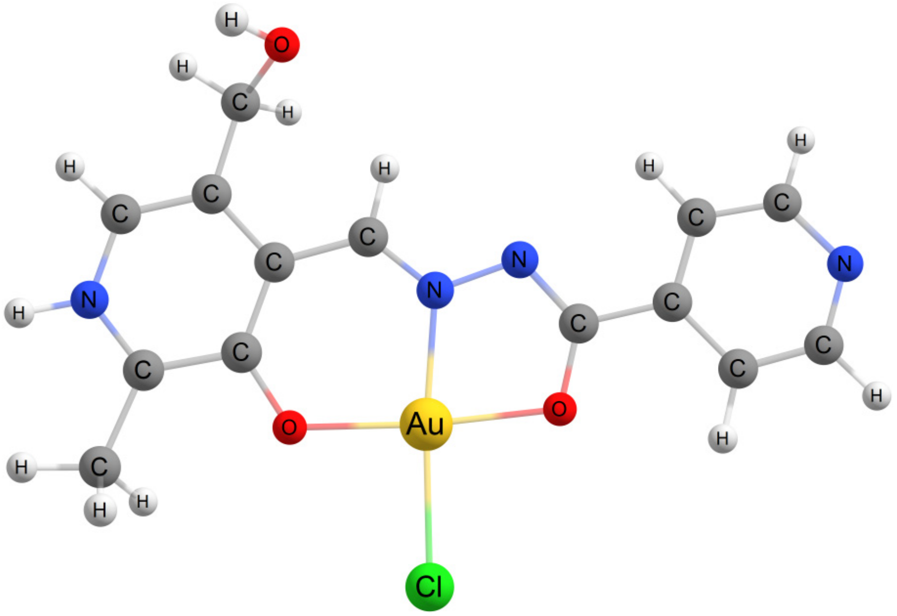

6	-4.093447000	-0.401178000	-0.035475000
6	-2.698549000	-0.117632000	-0.036092000
6	-2.277437000	1.236635000	-0.101556000
6	-3.260753000	2.262956000	-0.191509000
6	-4.583313000	1.920496000	-0.192561000
7	-4.942084000	0.618956000	-0.113265000
1	-5.386933000	2.634838000	-0.255178000
6	-4.612520000	-1.790212000	0.048887000
1	-4.255740000	-2.266311000	0.962536000
1	-4.234797000	-2.380713000	-0.786141000
1	-5.699543000	-1.807185000	0.037066000
8	-1.955816000	-1.196282000	0.024067000
6	-0.895568000	1.653269000	-0.091958000
1	-0.670619000	2.710181000	-0.098302000

6	-2.908579000	3.733226000	-0.302453000
1	-3.828974000	4.307151000	-0.414404000
1	-2.309513000	3.904260000	-1.196651000
8	-2.132312000	4.211276000	0.789187000
7	0.123581000	0.864377000	-0.050963000
7	1.394396000	1.359146000	-0.053039000
6	2.287882000	0.407867000	-0.024433000
8	2.001330000	-0.874527000	0.008028000
79	0.023876000	-1.124018000	0.013175000
17	0.116061000	-3.441095000	0.072039000
6	3.721645000	0.762662000	-0.029757000
6	4.135824000	2.092697000	-0.106853000
6	5.495778000	2.363061000	-0.109956000
7	6.438351000	1.418049000	-0.041869000
6	6.031653000	0.148950000	0.032260000
6	4.696250000	-0.230736000	0.040989000
1	3.416588000	2.894759000	-0.164290000
1	5.842347000	3.387505000	-0.170452000
1	6.807359000	-0.605049000	0.087276000
1	4.424560000	-1.273020000	0.102180000
1	-2.669405000	4.186678000	1.589328000
1	-5.933119000	0.403156000	-0.111989000

Monoprotonated complex, $[AuCl(HL)]^{+}$ 2, proton is bound with hydrazide nitrogen

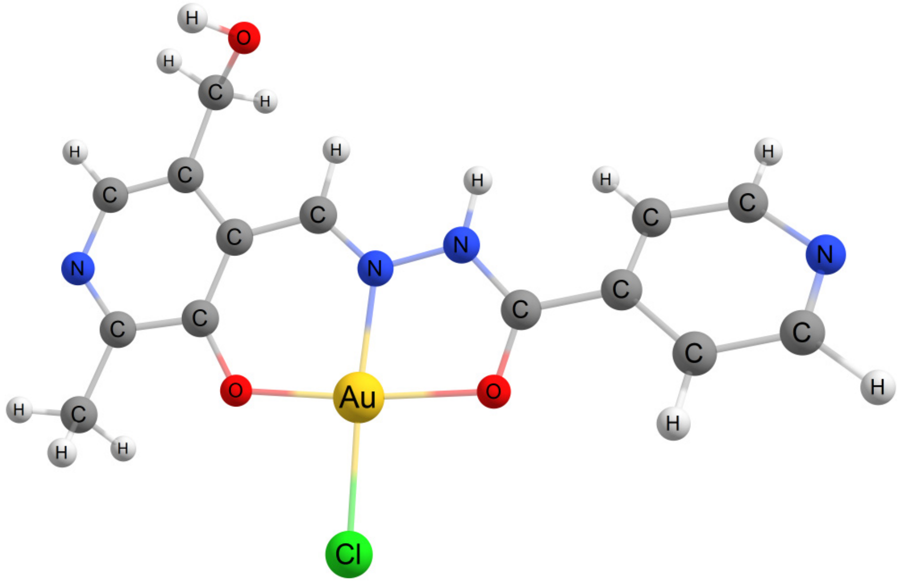

6	-4.143382000	-0.355408000	-0.033414000
6	-2.731455000	-0.086750000	-0.036213000
6	-2.306426000	1.264932000	-0.070047000
6	-3.303289000	2.285730000	-0.115751000
6	-4.619360000	1.901657000	-0.104759000
7	-5.023567000	0.617603000	-0.063914000
1	-5.403617000	2.647191000	-0.132778000

6	-4.623649000	-1.772924000	0.011528000
1	-4.256798000	-2.281870000	0.904352000
1	-4.255646000	-2.337601000	-0.846606000
1	-5.709942000	-1.784246000	0.011187000
8	-1.991362000	-1.180331000	-0.002996000
6	-0.935072000	1.677835000	-0.060480000
1	-0.722426000	2.740021000	-0.028344000
6	-2.968094000	3.756928000	-0.209292000
1	-3.898758000	4.325613000	-0.225563000
1	-2.443155000	3.968099000	-1.141770000
8	-2.103293000	4.223899000	0.827983000
7	0.064988000	0.863215000	-0.051714000
7	1.376211000	1.285174000	-0.027638000
6	2.324134000	0.348422000	-0.028400000
8	1.998228000	-0.883041000	-0.021200000
79	-0.024754000	-1.129751000	-0.016410000
17	0.029181000	-3.429345000	0.011238000
6	3.744459000	0.725361000	-0.028206000
6	4.192947000	1.934556000	-0.557552000
6	5.555160000	2.199302000	-0.518090000
7	6.453021000	1.360396000	0.001266000
6	6.011477000	0.203471000	0.499642000
6	4.674161000	-0.166024000	0.504668000
1	3.527150000	2.646274000	-1.022948000
1	5.935240000	3.126233000	-0.928087000
1	6.757264000	-0.462291000	0.914849000
1	4.364609000	-1.111739000	0.921852000
1	-2.554654000	4.124879000	1.673988000
1	1.567400000	2.277178000	0.047130000

Bis-protonated complex, $[AuCl(H_2L)]^{2+}$

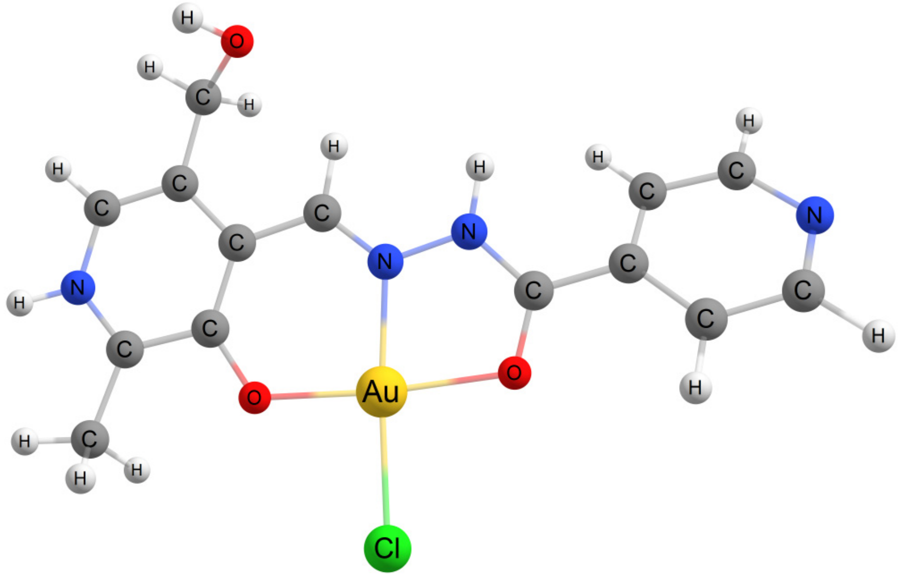

6	-4.098811000	-0.408164000	-0.043661000
6	-2.702780000	-0.108570000	-0.030378000
6	-2.298797000	1.251215000	-0.057614000
6	-3.289666000	2.273937000	-0.111117000
6	-4.607248000	1.915636000	-0.120407000
7	-4.950607000	0.607293000	-0.087620000
1	-5.420030000	2.621410000	-0.154336000
6	-4.603062000	-1.803296000	-0.005957000
1	-4.243113000	-2.304180000	0.893133000
1	-4.216230000	-2.361369000	-0.859045000
1	-5.689526000	-1.830376000	-0.021735000
8	-1.962868000	-1.192111000	0.006373000
6	-0.918875000	1.680240000	-0.043488000
1	-0.718410000	2.744314000	-0.004997000
6	-2.957479000	3.752946000	-0.194545000
1	-3.887132000	4.320976000	-0.171960000
1	-2.473182000	3.961964000	-1.148590000
8	-2.056978000	4.195506000	0.811227000
7	0.078702000	0.870955000	-0.038245000
7	1.385049000	1.294741000	-0.016002000
6	2.336783000	0.356621000	-0.025675000
8	2.009439000	-0.874511000	-0.022120000
79	0.004336000	-1.127401000	-0.010207000
17	0.074880000	-3.417250000	0.012232000
6	3.754714000	0.731019000	-0.031695000
6	4.197911000	1.953006000	-0.536980000
6	5.560730000	2.214850000	-0.505880000
7	6.462207000	1.362112000	-0.016403000
6	6.025891000	0.193806000	0.459190000
6	4.688473000	-0.174853000	0.470280000
1	3.529322000	2.677278000	-0.978119000
1	5.937768000	3.150946000	-0.897107000
1	6.775613000	-0.481933000	0.850099000
1	4.382686000	-1.129439000	0.869483000
1	-2.498967000	4.169814000	1.667726000
1	1.575694000	2.287456000	0.059534000
1	-5.940938000	0.383950000	-0.094023000

Table S14. Calculated IR spectra of different protonated species of complex formed by gold(III) and hydrazone derived from pyridoxal and isoniazid (**PL-INH**)

Deprotonated complex, [AuClL]		Monoprotonated complex 1, $[AuCl(HL)]^+$		Monoprotonated complex 2, $[AuCl(HL)]^+$		*Bis*-protonated complex, $[AuCl(H_2L)]^{2+}$	
Frequency, cm^{-1}	Relative intensity	Frequency, cm^{-1}	Relative intensity	Frequency, cm^{-1}	Relative intensity	Frequency, cm^{-1}	Relative intensity
25.234	0.005	25.7909	0.5011	27.436	0.3728	25.7816	2.2386
28.4357	0.0328	29.1851	4.7583	34.6869	0.0616	29.9603	1.8541
46.7445	1.938	51.4667	1.1232	56.9997	10.6151	56.8783	9.5267
63.7235	1.2059	64.5623	2.3746	61.3605	0.6202	62.6262	3.8075
75.8068	2.7038	78.6758	4.7726	74.6905	4.7075	73.495	3.1154
93.0604	0.0922	95.1959	2.0014	91.2427	0.235	93.5734	1.4026
104.3364	1.5697	105.1486	0.302	111.9386	1.7732	112.9285	1.8602
110.661	1.4969	111.6764	3.6807	113.384	3.1776	116.2858	5.9007
118.0337	3.3218	124.2903	1.3108	124.6729	14.7695	126.2376	6.8738
158.5044	0.2976	149.569	0.9839	156.2912	0.4915	144.6969	2.8736
174.3077	4.9522	174.1649	2.2357	167.6553	4.581	169.7349	3.8756
183.71	0.6742	182.1263	1.1961	177.3144	3.641	179.9165	2.7511
190.2579	1.7542	191.0754	1.0237	187.4333	3.4289	187.6066	3.839
203.2267	0.5421	207.3802	0.6718	196.637	2.6337	200.7883	5.2239
213.6169	1.359	213.19	2.6897	208.5171	4.2698	211.2957	6.3462
234.4157	5.1913	234.7293	1.8583	228.4917	6.0389	228.7371	3.7158
242.3978	14.1271	245.0301	11.4934	252.8347	12.4576	255.1092	10.6634
275.1414	2.4789	272.6038	7.1822	275.538	0.6446	274.7404	3.9972
307.2149	132.6844	310.6085	103.1665	322.2015	47.6087	325.3439	19.579
326.2706	70.873	334.9631	73.7484	334.2352	12.5553	339.732	12.687
342.8666	13.242	342.5924	18.4815	348.3277	43.1063	356.2834	41.1346
361.8599	72.9326	362.7549	77.6933	367.3489	171.2607	378.5724	162.7356
389.7496	0.0152	388.1953	0.0026	377.7151	5.8749	379.2868	9.9191
401.1603	4.2606	395.4016	9.0536	382.5082	0.2137	386.366	13.5687
414.2977	29.8611	415.8197	28.1183	393.2437	8.025	390.6222	8.8429
427.7643	1.9514	442.7101	5.4746	399.4135	5.9332	411.2613	7.0534
446.8648	30.6541	445.3722	18.0762	446.0432	35.4152	443.1355	27.4313
484.6092	20.5705	484.2598	15.0985	474.4636	32.0168	475.1933	32.1246
506.6761	6.9959	508.7276	9.0354	491.6914	14.2257	494.3538	7.1266
531.1007	12.1121	530.5372	12.1406	516.9223	95.0852	527.5215	101.9926
573.1589	10.5929	569.9554	2.0335	532.5081	22.7371	532.5665	29.5609
578.3782	25.4935	571.7317	9.4463	569.4083	10.5547	565.0687	8.8529
588.0752	29.5041	586.5779	8.8046	572.1875	43.5524	570.2265	14.9776
644.6772	42.9859	648.7096	13.9742	577.0581	46.5976	577.1397	17.5255
659.5016	9.6301	662.2956	16.4632	632.3129	58.1175	636.6498	24.1889
681.0447	3.0533	680.735	2.975	650.8869	13.3196	654.2616	19.3105
685.5987	25.913	687.165	27.6446	677.5473	1.8018	676.9925	2.0846
709.9443	46.4729	713.263	47.8733	685.7851	10.3106	688.328	8.7931
724.2883	119.0037	726.8449	115.2669	716.7702	75.0355	718.7266	67.0797
758.6513	0.8979	760.527	1.6427	721.0433	45.6343	723.4964	51.5842

768.2908	10.4063	767.3981	12.3137	759.2922	2.5254	761.0462	11.5723
777.0901	6.5682	777.7584	0.5471	763.2338	15.5522	761.8175	16.9145
804.6222	2.4777	792.8341	46.9401	774.9663	6.3275	778.3444	4.7025
868.3109	27.8206	838.7765	80.3899	802.0991	0.7575	797.1324	34.5858
897.374	0.0142	868.7225	25.6459	858.1057	25.594	845.3882	94.0483
919.0656	30.3012	896.7646	0.014	887.981	4.6036	856.8671	21.2165
943.0363	0.4728	923.8205	1.3855	920.8686	28.1823	885.5012	5.9362
956.4919	2.6757	943.7045	8.8832	942.7882	3.1932	928.0039	1.6757
964.194	20.9135	948.7987	6.9942	960.2538	2.6696	944.3385	1.3757
988.021	99.0099	965.7594	21.2731	970.6297	23.614	960.0748	4.5573
1005.9005	1.0904	1000.3005	89.3395	987.7392	107.5333	976.6998	19.9807
1012.44	0.842	1007.0569	0.9915	1004.1209	2.0564	1001.6506	62.6437
1018.8163	25.2974	1012.7972	0.896	1013.1509	0.0764	1004.3553	9.3225
1020.5884	0.0327	1021.5049	0.0042	1023.152	1.7924	1012.9122	0.2123
1028.6993	146.8453	1028.6048	121.2371	1028.7059	70.8564	1023.5898	1.4821
1054.2303	1.7841	1045.5214	156.2667	1044.5812	44.7059	1043.6115	38.157
1071.0244	20.1984	1058.2166	9.3182	1054.863	1.8743	1053.2331	150.1935
1089.1134	17.1726	1079.3752	30.0903	1070.5119	36.4706	1059.1013	10.9647
1094.9922	19.2006	1089.5826	8.715	1096.9111	24.6847	1077.1515	50.5192
1112.5323	0.2242	1094.5212	58.5693	1126.1366	1.8679	1097.2498	29.5832
1162.4045	28.2681	1113.9872	0.1414	1139.2553	16.0049	1127.0472	2.6504
1199.8916	78.2306	1170.0009	63.4359	1184.6739	20.7279	1139.7345	21.6335
1210.9551	222.0794	1206.6125	12.9535	1197.4184	262.4239	1185.0499	24.3526
1239.7413	6.3291	1221.55	220.0644	1205.8372	23.892	1210.2467	93.4085
1271.923	10.9713	1240.5211	8.5627	1248.6067	0.2557	1212.931	153.5637
1290.7345	183.6294	1269.0948	66.1766	1268.7367	29.7114	1249.1931	1.3644
1313.8037	33.6744	1273.529	84.0027	1294.9129	146.0525	1268.4797	36.4896
1322.9928	117.2383	1322.883	5.1542	1309.9164	68.1239	1269.5784	120.2056
1335.3384	185.6072	1330.3139	107.1675	1323.7523	160.913	1317.7421	80.0155
1360.7518	49.4772	1349.6012	215.9752	1334.8938	156.3798	1335.0247	116.2449
1370.1608	10.4995	1362.9327	157.997	1366.9764	24.2285	1340.7034	220.1215
1392.4778	133.6368	1376.5108	20.3876	1374.3173	17.8096	1367.9302	22.38
1406.2334	93.4305	1394.0202	147.102	1386.602	64.7543	1382.8585	99.4278
1411.0356	43.7954	1408.037	100.5121	1407.0177	67.2843	1390.9144	41.5668
1420.056	37.3025	1413.5022	68.3857	1411.7709	4.2769	1406.5305	84.6292
1441.4403	34.132	1428.5825	0.7993	1420.9861	248.529	1417.9324	75.4091
1464.8216	24.0521	1439.2068	127.7159	1442.9953	42.985	1424.382	277.9275
1466.696	12.9838	1441.0409	17.0184	1464.0591	20.5289	1436.3918	46.4866
1484.4706	0.406	1452.5898	17.2585	1466.9238	11.9881	1444.1347	58.2644
1511.4655	6.8765	1483.5307	84.593	1481.8412	19.6399	1450.6801	18.1352
1527.9143	90.975	1506.8754	62.1254	1501.7131	276.7395	1484.1661	29.605
1547.6089	175.4316	1512.7694	194.8291	1510.1026	95.1683	1497.8937	28.2637
1555.7466	409.2188	1530.5032	62.5926	1528.3657	17.5683	1511.2638	150.228
1600.9509	29.0366	1537.3157	913.9686	1550.9473	200.5191	1521.0975	247.1019
1607.3552	72.055	1601.5583	19.5639	1595.6693	523.6791	1533.2596	219.8729
1633.533	157.4605	1616.7061	59.67	1599.537	73.1049	1596.5214	697.595

1652.3646	90.137	1634.8639	56.6169	1605.9305	72.7645	1601.7871	53.2729
3035.7151	23.1871	1654.4142	16.592	1633.0606	2.1181	1619.4743	14.3173
3036.9091	69.332	1671.4424	63.2817	1665.7776	119.0373	1631.2484	10.3029
3077.4837	28.8951	3048.8531	2.0873	3038.5267	13.7095	1665.1815	68.3969
3081.5561	16.6951	3051.1135	48.7685	3043.7089	58.4046	1683.9327	37.0949
3135.0695	22.3581	3092.6891	19.0749	3081.6607	26.6635	3049.9363	2.2248
3158.8913	48.2008	3103.4776	0.5564	3085.7377	12.9396	3057.3096	33.2516
3162.7535	25.3185	3138.0642	14.6299	3139.8591	17.9461	3097.251	19.1149
3163.8754	42.3783	3161.7605	44.5016	3170.5215	81.731	3105.2606	0.0426
3196.1476	29.7964	3165.6153	22.6809	3172.4863	32.3366	3141.6091	12.26
3219.5038	2.7506	3204.9938	48.5017	3172.5185	31.1527	3172.3466	132.7629
3219.9951	3.545	3220.8959	2.7319	3176.0064	10.4864	3174.6032	26.4247
3791.7181	68.6433	3221.4451	3.8439	3207.2216	3.0449	3178.1034	8.9881
		3251.4903	4.78	3222.7063	2.7167	3208.2745	2.8533
		3542.8436	364.4055	3562.0762	182.7717	3223.9175	2.9586
		3790.7043	91.1436	3792.613	77.5165	3258.4556	10.2695
						3528.6563	337.407
						3554.4004	198.7356
						3792.9931	99.4694

Table S15. Calculated TD-DFT spectra of different protonated species of complex formed by gold(III) and hydrazone derived from pyridoxal and isoniazid (**PL-INH**)

Deprotonated complex, [AuClL]		Monoprotonated complex 1, $[AuCl(HL)]^+$		Monoprotonated complex 2, $[AuCl(HL)]^+$		*Bis*-protonated complex, $[AuCl(H_2L)]^{2+}$	
λ, nm	oscillator strength	λ, nm	oscillator strength	λ, nm	oscillator strength	λ, nm	oscillator strength
461.49	0.0001	420.98	0.0002	523.28	0.0001	448.8	0.0001
368.06	0.1511	372.57	0.2854	381.75	0.0681	379.6	0.0183
367.15	0.0253	346.9	0	371.74	0.0001	366.96	0
318.76	0.0241	328.97	0.0479	357.25	0.0524	361.84	0.1542
305.89	0.0001	316.98	0.0001	315.03	0.0005	299.64	0.0002
299.08	0.3955	304.39	0.3874	313.45	0.0034	296.66	0.0057
295.24	0.0482	282.82	0.0656	301.89	0.004	288.54	0.0077
279.41	0.0297	277.18	0.0021	287.44	0.0182	283.74	0.1506
266.36	0.0029	269.54	0.0009	282.97	0.2346	281.19	0.1866
263.53	0.1935	267.79	0.0553	279.27	0.1848	270.81	0.025
261.14	0.2276	259.53	0.2118	272.04	0.4007	264.44	0.0063
253.13	0.0967	252.56	0.0244	269.12	0.0678	260.17	0.3245
246.61	0.0022	252.55	0.0008	257.02	0.0126	255.71	0.242
242.21	0.3022	248.05	0.0005	253.39	0.0386	249.76	0.0037
239.56	0.0001	239.49	0.1086	244.49	0.1984	241.69	0.0898
234.01	0.0962	237.06	0.3734	238.73	0.0036	237.28	0.2572
229.83	0.0316	233.58	0.0769	237.69	0.0589	234.27	0.0761
227.02	0.0023	231.05	0.0105	233.56	0.0955	230.51	0.0103
224.27	0.0592	229.69	0.0297	225.22	0.1952	226.74	0.0257
220.64	0.0387	220.78	0.0233	221.66	0.0188	223.81	0.0548
218.38	0.0064	220.46	0.0083	221.52	0.0221	222.76	0.0028
217.7	0.0008	219.45	0.0005	220.63	0.0973	222.63	0.0032
216.17	0.0579	218.83	0.0441	216.73	0.0052	220.45	0.0193
211.51	0.1382	218.16	0.0179	212.56	0.0647	219.38	0.0406
210.56	0.0654	216.11	0.0493	211.45	0.0011	213.97	0.1078
208.32	0.0101	212.05	0.0017	210.42	0.1053	210.94	0.2199
208.02	0.0254	209.85	0.2567	207.87	0.059	209.11	0.0742
206.11	0.0313	208.61	0.0405	204.83	0.0187	205.47	0.2237
205.16	0.2111	204.87	0.0252	204.03	0.0207	198.77	0.0478
203.94	0.0858	202	0.0244	203.9	0.0366	198.48	0.0195

Table S16. Shape of selected frontier molecular orbitals of gold(III) complexes with hydrazones derived pyridoxal obtained by DFT calculations

**PL-F3H**						
[AuClL]	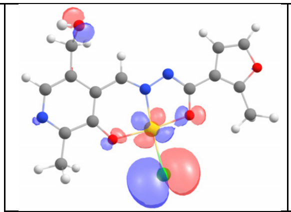	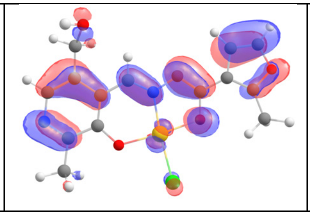	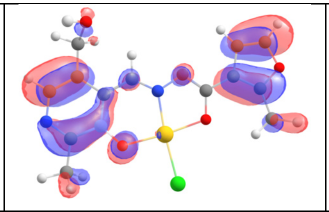	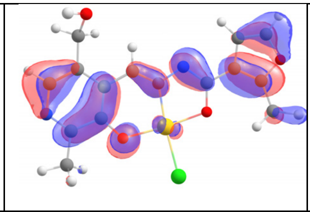	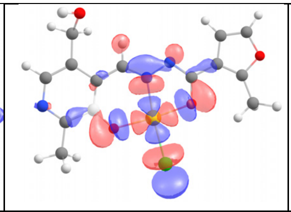	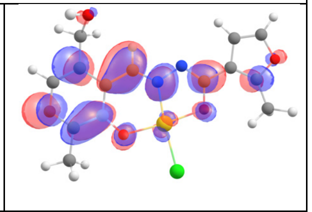
	HOMO-5(88)	HOMO-2(91)	HOMO-1(92)	HOMO(93)	LUMO(94)	LUMO+1(95)
[AuCl(HL)]$^{+}$ 2	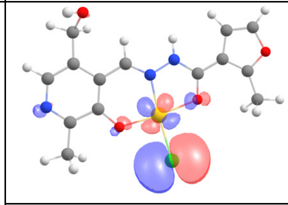	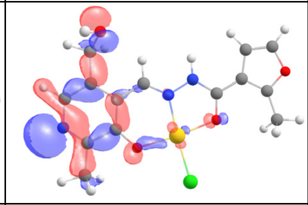	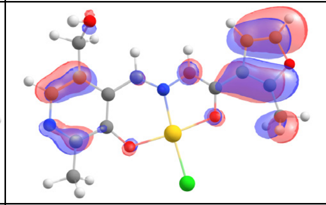	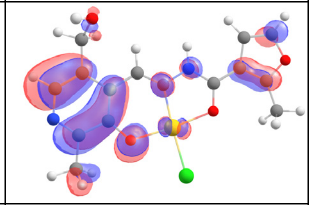	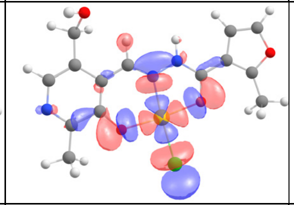	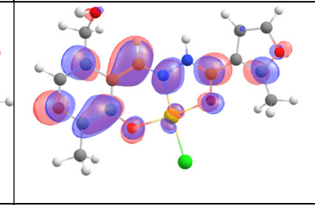
	HOMO-5(88)	HOMO-2(91)	HOMO-1(92)	HOMO(93)	LUMO(94)	LUMO+1(95)
$[AuCl(H_2L)]^{+2}$	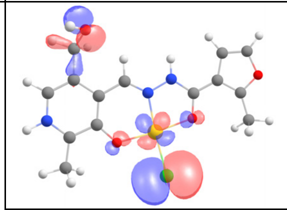	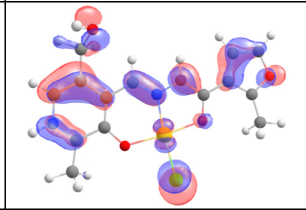	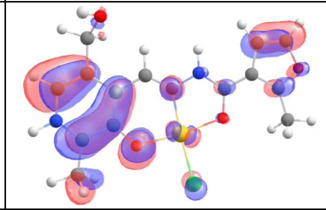	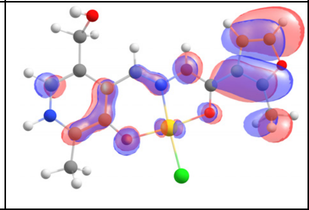	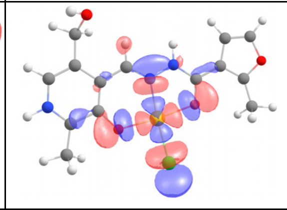	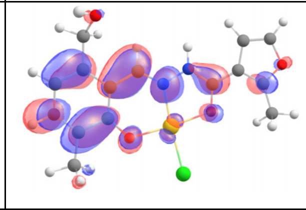
	HOMO-3(90)	HOMO-2(91)	HOMO-1(92)	HOMO(93)	LUMO(94)	LUMO+1(95)
**PL-F2H**						
[AuClL]	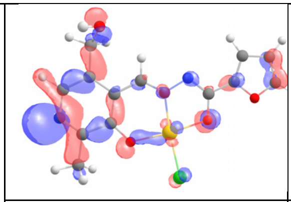	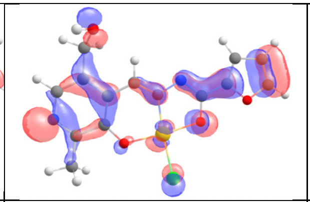	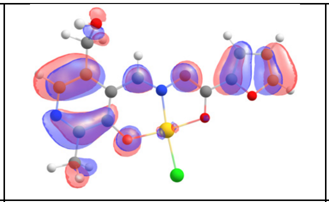	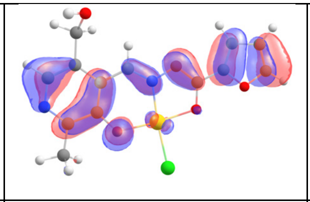	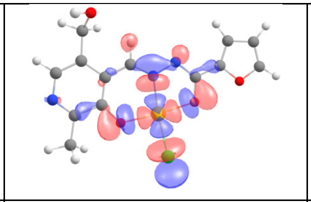	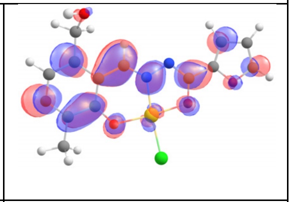
	HOMO-3(86)	HOMO-2(87)	HOMO-1(88)	HOMO(89)	LUMO(90)	LUMO+1(91)

$[AuCl(HL)]^{+}$ 2	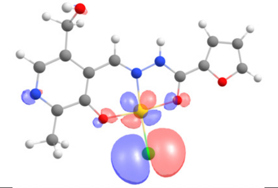	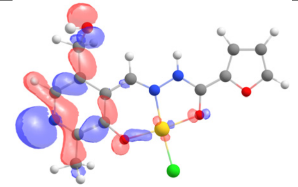	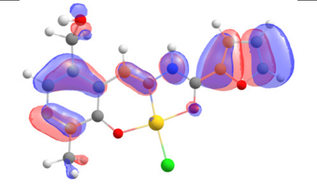	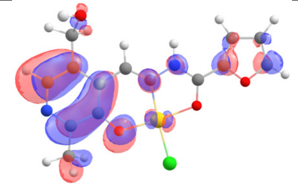	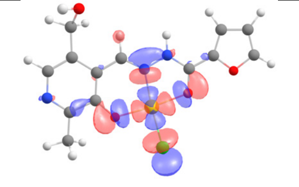	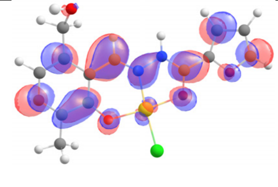
	HOMO-5(84)	HOMO-2(87)	HOMO-1(88)	HOMO(89)	LUMO(90)	LUMO+1(91)
$[AuCl(H_2L)]^{+2}$	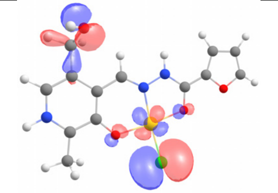	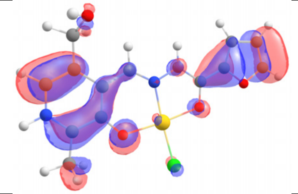	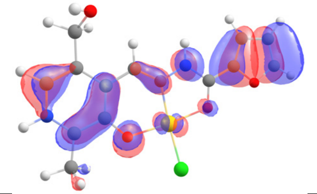	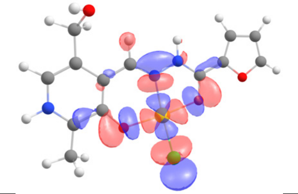	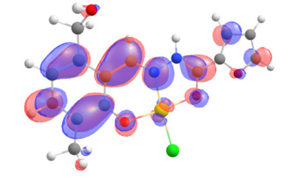	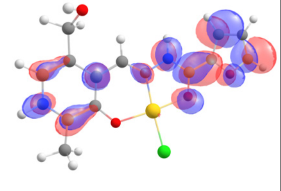
	HOMO-3(86)	HOMO-1(88)	HOMO(89)	LUMO (90)	LUMO+1(91)	LUMO+2(92)
**PL-T3H**						
[AuClL]	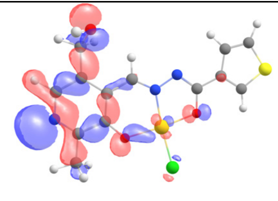	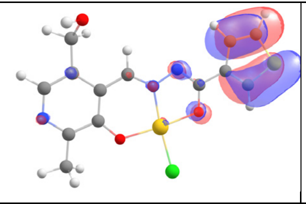	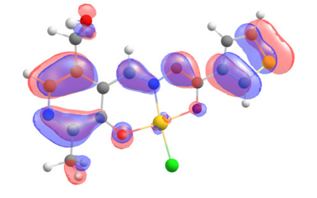	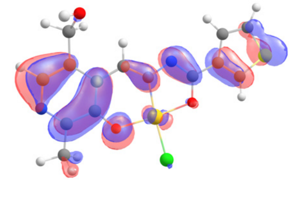	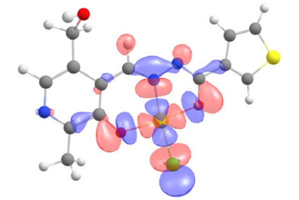	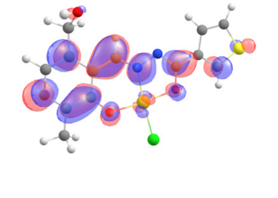
	HOMO-3(90)	HOMO-2(91)	HOMO-1(92)	HOMO(93)	LUMO(94)	LUMO+1(95)
$[AuCl(HL)]^{+}$ 2	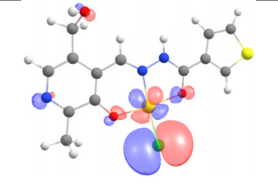	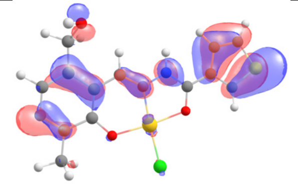	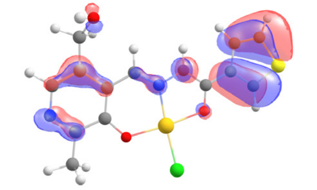	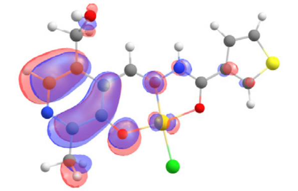	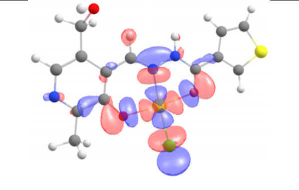	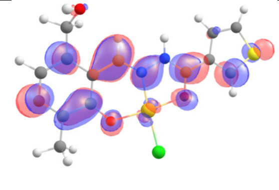
	HOMO-3(87)	HOMO-2(91)	HOMO-1(92)	HOMO(93)	LUMO(94)	LUMO+1(95)
$[AuCl(H_2L)]^{+2}$	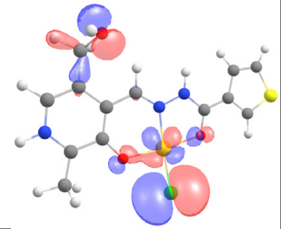	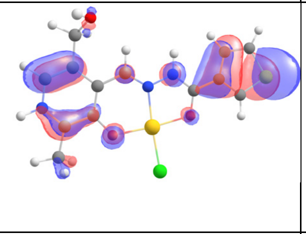	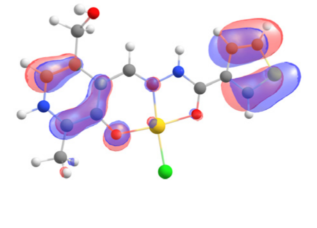	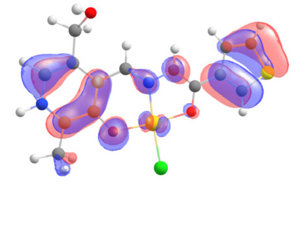	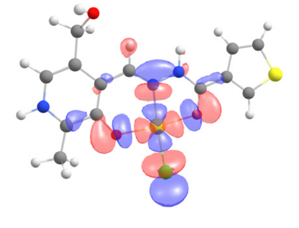	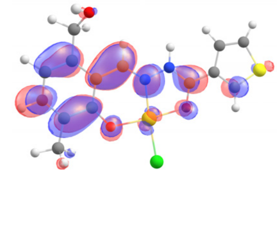
	HOMO-4(89)	HOMO-2(91)	HOMO-1(92)	HOMO(93)	LUMO(94)	LUMO+1(95)
**PL-T2H**						

[AuClL]	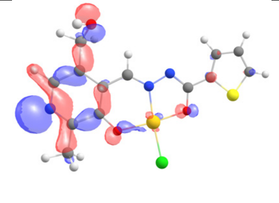	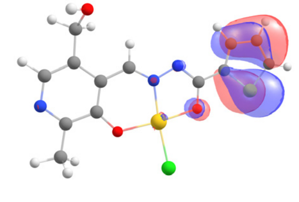	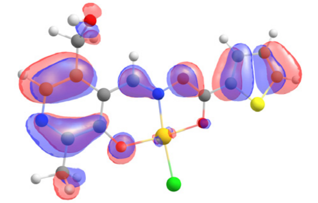	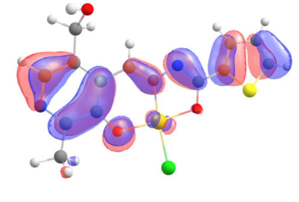	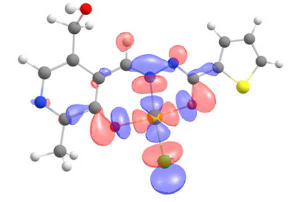	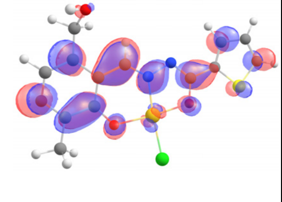
	HOMO-3(90)	HOMO-2(91)	HOMO-1(92)	HOMO(93)	LUMO(94)	LUMO+1(95)
$[AuCl(HL)]^{+}$ 2	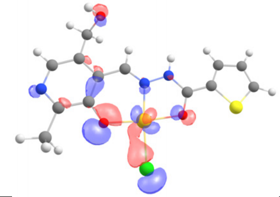	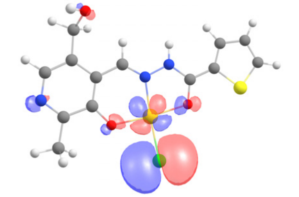	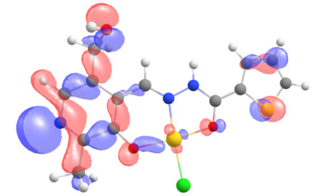	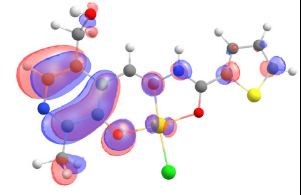	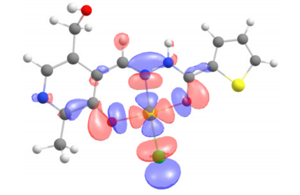	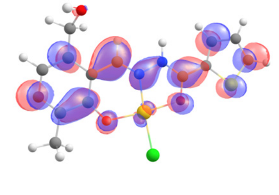
	HOMO-9(84)	HOMO-6(87)	HOMO-2(91)	HOMO(93)	LUMO(94)	LUMO+1(95)
$[AuCl(H_2L)]^{+2}$	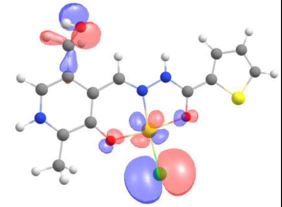	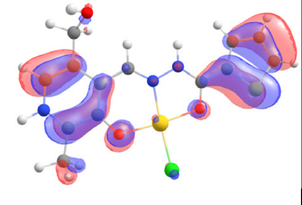	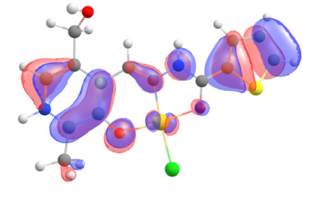	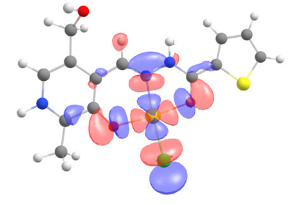	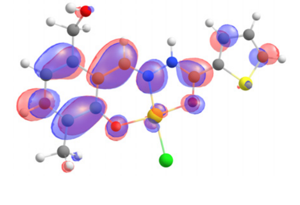	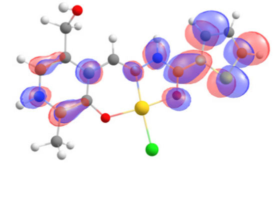
	HOMO-4(89)	HOMO-1(92)	HOMO(93)	LUMO(94)	LUMO+1(95)	LUMO+2(96)
**PL-INH**						
[AuClL]	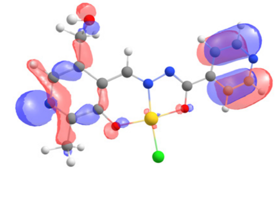	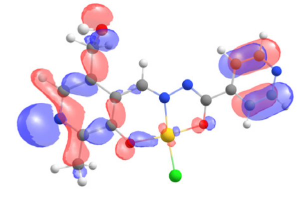	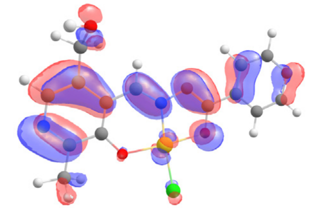	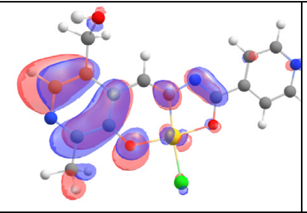	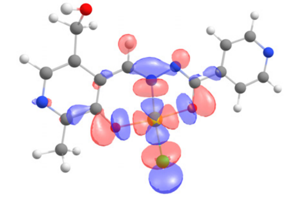	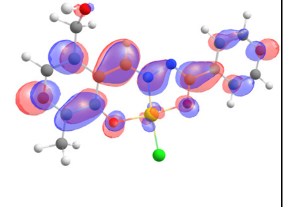
	HOMO-3(89)	HOMO-2(90)	HOMO-1(91)	HOMO(92)	LUMO(93)	LUMO+1(94)
$[AuCl(HL)]^{+}$ 2	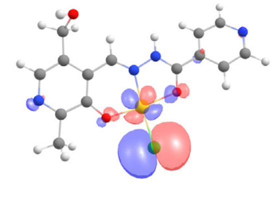	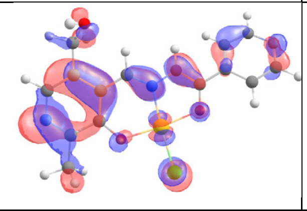	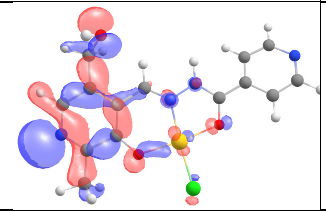	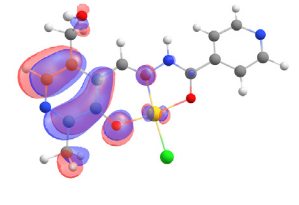	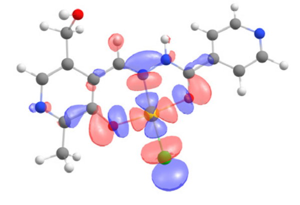	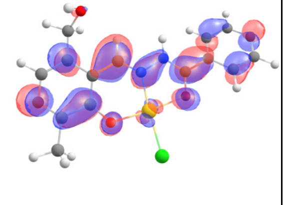
	HOMO-6(86)	HOMO-2(90)	HOMO-1(91)	HOMO(92)	LUMO(93)	LUMO+1(94)

$[AuCl(H_2L)]^{+2}$	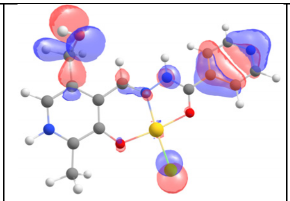	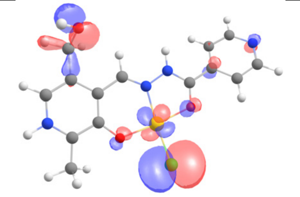	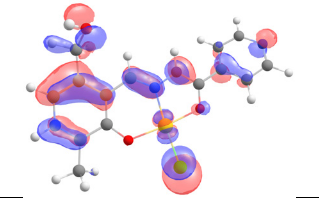	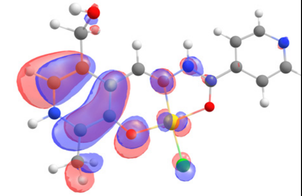	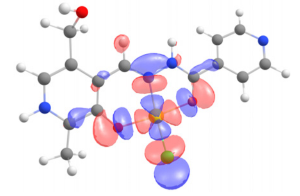	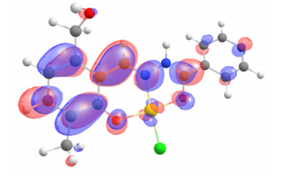
	HOMO-6(86)	HOMO-4(88)	HOMO-3(89)	HOMO(92)	LUMO(93)	LUMO+1(94)
